# Time-course global proteome analyses reveal an inverse correlation between Aβ burden and immunoglobulin M levels in the *APP^NL-F^* mouse model of Alzheimer disease

**DOI:** 10.1371/journal.pone.0182844

**Published:** 2017-08-23

**Authors:** Hansen Wang, Declan Williams, Jennifer Griffin, Takashi Saito, Takaomi C. Saido, Paul E. Fraser, Ekaterina Rogaeva, Gerold Schmitt-Ulms

**Affiliations:** 1 Tanz Centre for Research in Neurodegenerative Diseases, University of Toronto, Toronto, Canada; 2 Laboratory for Proteolytic Neuroscience, RIKEN Brain Science Institute, Hirosawa, Wako-shi, Saitama, Japan; 3 Department of Medical Biophysics, University of Toronto, Toronto, Canada; 4 Department of Medicine (Neurology), University of Toronto, Toronto, Canada; 5 Department of Laboratory Medicine & Pathobiology, University of Toronto, Toronto, Canada; Nathan S Kline Institute, UNITED STATES

## Abstract

Alzheimer disease (AD) stands out amongst highly prevalent diseases because there is no effective treatment nor can the disease be reliably diagnosed at an early stage. A hallmark of AD is the accumulation of aggregation-prone amyloid β peptides (Aβ), the main constituent of amyloid plaques. To identify Aβ-dependent changes to the global proteome we used the recently introduced *APP*^*NL-F*^ mouse model of AD, which faithfully recapitulates the Aβ pathology of the disease, and a workflow that interrogated the brain proteome of these mice by quantitative mass spectrometry at three different ages. The elevated Aβ burden in these mice was observed to cause almost no changes to steady-state protein levels of the most abundant >2,500 brain proteins, including 12 proteins encoded by well-confirmed AD risk loci. The notable exception was a striking reduction in immunoglobulin heavy mu chain (IGHM) protein levels in homozygote *APP*^*NL-F/NL-F*^ mice, relative to *APP*^*NL-F/wt*^ littermates. Follow-up experiments revealed that IGHM levels generally increase with age in this model. Although discovered with brain samples, the relative IGHM depletion in *APP*^*NL-F/NL-F*^ mice was validated to manifest systemically in the blood, and did not extend to other blood proteins, including immunoglobulin G. Results presented are consistent with a cause-effect relationship between the excessive accumulation of Aβ and the selective depletion of IGHM levels, which may be of relevance for understanding the etiology of the disease and ongoing efforts to devise blood-based AD diagnostics.

## Introduction

Alzheimer disease (AD) is the most prevalent neurodegenerative disease world-wide. In contrast to other common diseases, there is no prevention, cure or even slowing treatment for AD. Age represents the main risk factor for acquiring AD, its prevalence doubling every five to ten years—dependent on region—in individuals older than 65 years [1]. The disease is characterized by an extended prodromal phase that may last more than a decade and precedes the manifestation of increasingly debilitating symptoms. Individuals at the prodromal AD phase represent the most meaningful AD diagnosis target group, because future AD therapeutics are expected to be most effective when administered early. The ability to distinguish early manifestations of AD from other dementias is of utmost importance already today for the classification of subjects enrolled in AD trials. Currently, the disease cannot be reliably detected at this early stage and, even when more severe symptoms manifest, a firm diagnosis is often only made postmortem.

The concomitant accumulation of extracellular aggregates of amyloid β peptides (Aβ) and intracellular deposits of the tau protein is the most widely accepted neuropathological hallmark that distinguishes AD from other neurodegenerative diseases [2]. In inherited forms of the disease, an increased propensity of neurons to release aggregation-prone Aβ peptides represents the critical first event that sets off the disease [3]. The Aβ pathology also plays a central role in late-onset AD, where the dosage of the apolipoprotein E (*APOE)* epsilon 4 allele was identified as a major AD risk factor that impacts Aβ uptake and clearance [4]. In addition to *APOE* and three causal early onset AD genes (*APP*, *PSEN1*, *PSEN2*), genome-wide studies have identified variations in 24 loci that are associated with sporadic late onset AD, strongly implicating inflammation pathways in the molecular etiology of the disease [5].

Several mouse models, which recapitulate aspects of AD have been generated in the past twenty years [6]. None of the existing models capture all aspects of the disease. Early attempts to model the Aβ pathology of AD in mice were based on random genomic insertions of transgenes coding for the human amyloid precursor protein (APP) carrying mutations known to cause inherited forms of AD [7–10]. The endoproteolytic cleavage of APP by specific secretases causes the release of Aβ peptides in humans and mice [11]. An increasing awareness of inadvertent gene dosage, genomic insertion and unspecific promoter effects, combined with concern over unpredictable consequences of the unnatural release of extracellular and intracellular non-Aβ APP fragments that accompany the Aβ release, prompted the refinement of AD modeling strategies. Recently, a next-generation AD mouse model was presented, which circumvented the aforementioned confounders by a knock-in strategy that introduced Swedish (K670N/L671M) and Iberian/Beyreuther (I716F) mutations into the endogenous mouse *APP* gene, whose Aβ sequence was ‘humanized’ by the exchange of three codons within the Aβ coding sequence [12]. Homozygote *APP*^*NL-F/NL-F*^ mice exhibit increased Aβ levels at 6 months of age and eventually develop granular Aβ deposits that mimic human AD amyloid plaques in appearance and in their preferential composition of Aβ1–42 over Aβ1–40.

Here, we describe in-depth analyses of the brain proteome of *APP*^*NL-F/NL-F*^ mice in the search for changes to steady-state levels of individual proteins. We document that increased production of Aβ1–42 in *APP*^*NL-F/NL-F*^ mice has no discernible effect on the steady-state levels of more than 2,500 proteins, which were reliably quantified at time points that demark different stages of AD-like pathology in these mice. However, we report on a striking suppression of immunoglobulin heavy constant mu (IGHM) chain levels coinciding with increased Aβ burden. Follow-up validation work conclusively revealed that the relative reduction in IGHM levels we observed in *APP*^*NL-F/NL-F*^ mice occurred systemically in the blood rather than in the brain, the tissue known to endoproteolytically release the highest levels of Aβ from the *APP*^*NL-F*^ knock-in expression product. We further document that the depletion does not extend to other blood proteins, including immunoglobulin G antibodies. The data are discussed in the context of observations by others that have explored connections between the pathobiology of Aβ and immunoglobulins.

## Materials and methods

### Antibodies

Primary antibodies used in this study were the anti-Aβ1–16 (6E10) (catalog number SIG-39320, BioLegend, San Diego, CA, USA), anti-phospho-tau antibodies AT8 and AT180 targeting pSer202/pThr205 (catalog number MN1020, Thermo Fisher Scientific, Waltham, MA, USA) and pThr231 epitopes (catalog number MN1040, Thermo Fisher Scientific), respectively, the polyclonal rabbit anti-human tau antibody (K9JA) (catalog number A0024, Dako Canada Inc., Mississauga, ON, Canada), anti-mouse IgM (μ-chain specific) peroxidase conjugate (catalog number A8786, Sigma-Aldrich, St. Louis, MO, USA), goat anti-mouse IgG (H + L)-HRP conjugate (catalog number 170–6516, Bio-Rad Laboratories, Mississauga, ON, Canada), and the anti-beta actin (8H10D10) mouse monoclonal antibody (catalog number 3700, Cell Signaling Technology, Danvers, MA, USA).

### Mouse husbandry, breeding and genotyping

All animal protocols were in accordance with the Canadian Council on Animal Care and approved by the Animal Care and Use Committees at the University of Toronto and the University Health Network. The *APP* knock-in mouse line was originally from Dr. Takaomi C. Saido (RIKEN Brain Science Institute, Japan) [12]. To minimize inadvertent mouse strain effects, homozygote *APP*^*NL-F/NL-F*^ and heterozygote *APP*^*NL-F/wt*^ mice were produced by breeding heterozygote parents on a C57BL/6 genomic background, leading to *wt/wt*, *NL-F/NL-F* or *NL-F/wt* offspring in litters, which were genotyped with respect to their APP locus by an optimized APP^NL-F^-specific tail clip genotyping assay. More specifically, the mice were genotyped by polymerase chain reaction (PCR) using genomic DNA isolated from tail clippings. DNA samples (20 ng/reaction) were amplified using a cocktail of the following primers: 5’-ATCTCGGAAGTGAAGATG-3’, 5’-ATCTCGGAAGTGAATCTA-3’, 5’-TGTAGATGAGAACTTAAC-3’ and 5’-CGTATAATGTATGCTATACGAAG-3’. The PCR conditions were 94°C for 5 min, followed by 40 cycles of 94°C for 45 s, 54°C for 45 s, 72°C for 60 s, and 7 min at 72°C. The PCR products were analyzed by electrophoresis at 100 V in 1.2% agarose gels in Tris-Borate-EDTA (TBE) buffer.

TgCRND8 mice expressing the Swedish and Indiana mutations of human APP under the control of the hamster prion protein promoter were maintained on a hybrid C57/Bl6K:C3H genetic background [13]. The transgene was maintained in a hemizygous state by breeding positive males to non-transgenic females and genotyping was performed by dot blot. Mice were genotyped by dot blot. For whole blood collection, mice were anaesthetized with sodium pentobarbital (30–40 mg/kg). Once anaesthetic plane was achieved, mice were bled by cardiac puncture followed by tissue collection.

All animal studies followed the University of Toronto Animal Care Policies and Guidelines and were approved by the University Animal Care Committees of the University of Toronto and University Health Network.

### Mouse tissue preparation

Mice were euthanized and the brains were rapidly extracted from the skulls. Half-brains were homogenized in SDS-containing Lysis Buffer (2% SDS, 62.5 mM HEPES/NaOH, pH 8.0; preheated to 90°C), with the aid of 1.0 mm zirconia beads and the Mini-BeadBeater-8 (Biospec Products Inc., Oklahoma, USA). Following three one-minute cycles of bead beating, brain lysates were further incubated at 90°C to deactivate residual enzymatic activities in the extracts. The mouse blood was collected during transcardiac perfusion and solubilized in SDS-containing Lysis Buffer assisted by sonication with a Sonic Dismembrator device (Model 500, Thermo Fisher Scientific). For mouse serum preparation, blood was collected saphenously or transcardially and clotted in microvettes (CB300 Z, clotting/activator serum) (catalog number 16.440.100, Sarstedt, Nürnbrecht, Germany) at 4°C, followed by centrifugation (10,000 g, 5 min). Protein levels of the brain or blood samples were adjusted by bicinchoninic acid (BCA) colorimetric assay (Thermo Fisher Scientific). Identical volumes of serum samples were used for further analyses.

### Sample preparation for global proteome analyses

Protein precipitation, denaturation, alkylation and trypsin digestion of the brain lysate samples were performed as previously described [14]. Briefly, following homogenization, 5 mM tris(2-carboxyethyl) phosphine (TCEP) was added to homogenates and vials were heated at 60°C for 30 minutes then allowed to incubate for 1 hour in the presence of 10 mM 4-vinylpyiridine (4-VP) to facilitate reduction and alkylation. Next, homogenates were acetone-precipitated and precipitates were washed with 90% acetone before they were resolubilized in 9 M urea. Subsequently, samples were diluted with 100 mM tetraethylammonium bicarbonate (TEAB), pH 8, and digested with side chain-modified porcine trypsin (Thermo Fisher Scientific) overnight at 37°C. The resulting peptide mixtures were reacted with separate amine-modifying tandem mass tags (TMTs) from 6-plex reagent sets following the manufacturer’s protocol (Thermo Fisher Scientific).

### Quantitative mass spectrometry

**Fig 1:**
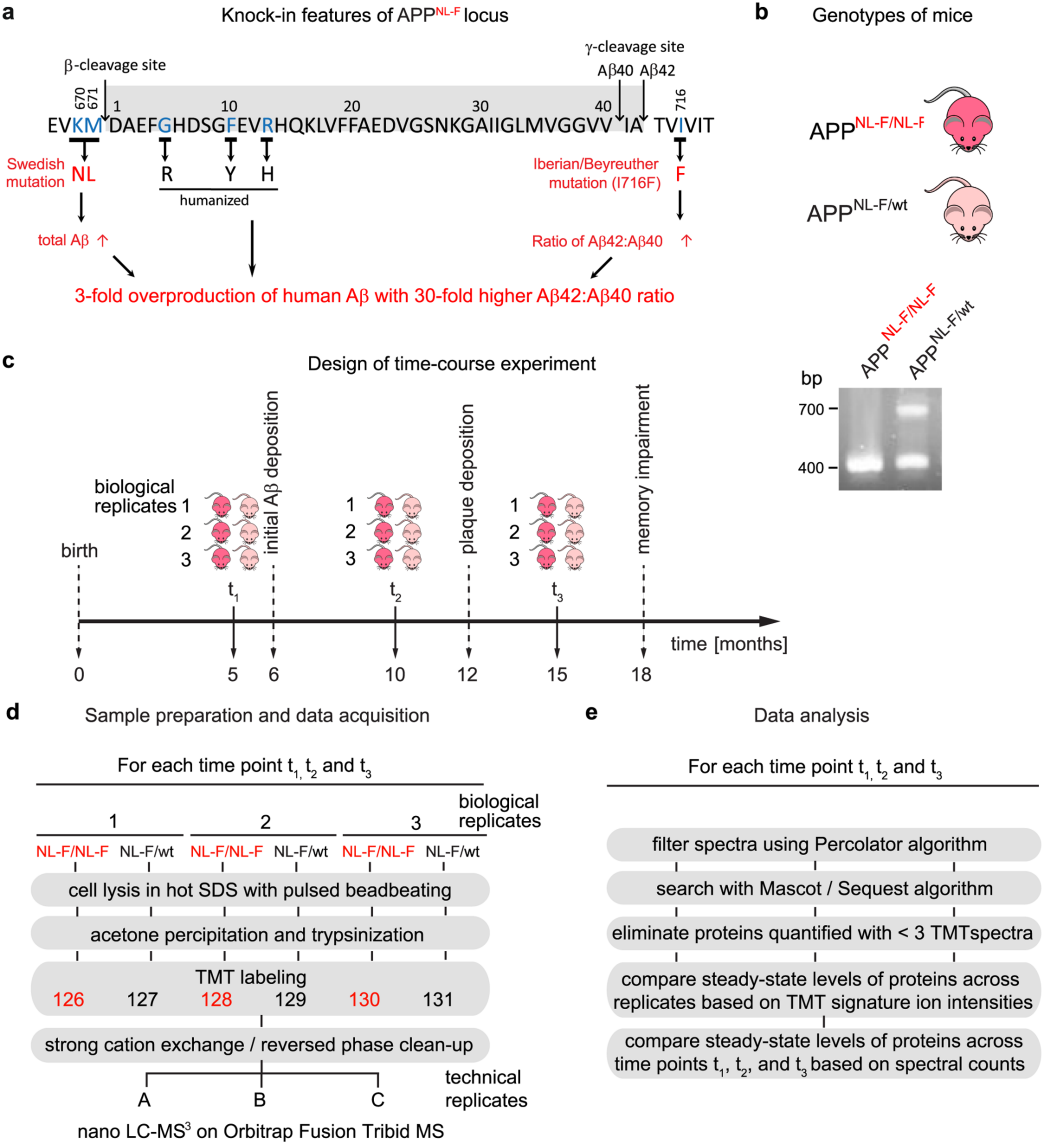
Time-course study design for comparing global proteomes of *APP*^*NL-F/NL-F*^ and *APP*^*NL-F/WT*^ mice. (**a**) Schematic depicting mutations present within the Aβ coding sequence or flanking it in the *APP*^*NL-F/NL-F*^ mouse model. The graph also depicts the predicted consequences of the mutations, i.e., (i) Swedish mutations lead to increased levels of Aβ compared to wild-type (wt) levels by approximately three-fold; (ii) the Aβ sequence was humanized by replacing three amino acids, and (iii) isoleucine at position Aβ45 was replaced by phenylalanine to increase the Aβ42:Aβ40 ratio approximately 30-fold. Design elements of this panel were adapted from a similar graph in the Supplementary Material section of Saito et al. [12]. (**b**) Genotypes of the mice used for the global proteome analyses. Representative genomic PCR results depicting amplification products of expected band sizes for the *APP*^*NL-F/NL-F*^ and *APP*^*NL-F/WT*^ alleles. (**c**) Design of the global proteome time-course analysis. Brains from three sex-matched (female) *APP*^*NL-F/NL-F*^ and *APP*^*NL-F/wt*^ mice were collected at 5, 10 and 15 months of age. (**d**) Workflow depicting sample preparation and data acquisition steps. Briefly, brains were rapidly dissected and homogenized in a bead beater in the presence of preheated (90°C) 2% SDS. Proteins were acetone precipitated, trypsinized and labeled with TMT reagents before deep proteome analysis on an Orbitrap Fusion Tribrid mass spectrometer. (**e**) Schematic depiction of post-acquisition data processing workflow.

The mass spectrometry procedure was similar to a previously described methodology [14]. Briefly, TMT-labeled peptide mixtures purified on C18 or SCX Bond Elut OMIX pipette tips (Agilent, Santa Clara, CA, USA) were subjected to reversed phase separation on a 25 cm nanobore Acclaim PepMap column (RSLCC18, 2 μm bead size, 100 Å pore size, 75 μm inner diameter) (Dionex, Oakville, ON, Canada) using a 4 h linear gradient of 2% to 95% acetonitrile in 0.1% formic acid, controlled by a split-free nanoflow liquid chromatography system (Easy-nLC 1100, Thermo Fisher Scientific), at a flow rate of 300 nL/min. The column eluent was sent to a 10 μm emitter tip for micro-ion spray ionization, and positively charged peptides were analyzed by an Orbitrap Fusion Tribrid mass spectrometer using a data-dependent acquisition method. This method included (i) a high resolution orbitrap survey scan, (ii) fragmentation of the most intense parent ions by CID for the identification of peptides in the ion trap, and (iii) detection of TMT signature ions from the 10 most intense peptide fragments upon their high-energy collision with nitrogen. Technical replicates of the LC-MS analysis were acquired from SCX and C18 pipette tip eluates of all three sample mixtures (Fig 1d).

### Post-acquisition processing of mass spectrometry data

**Fig 2:**
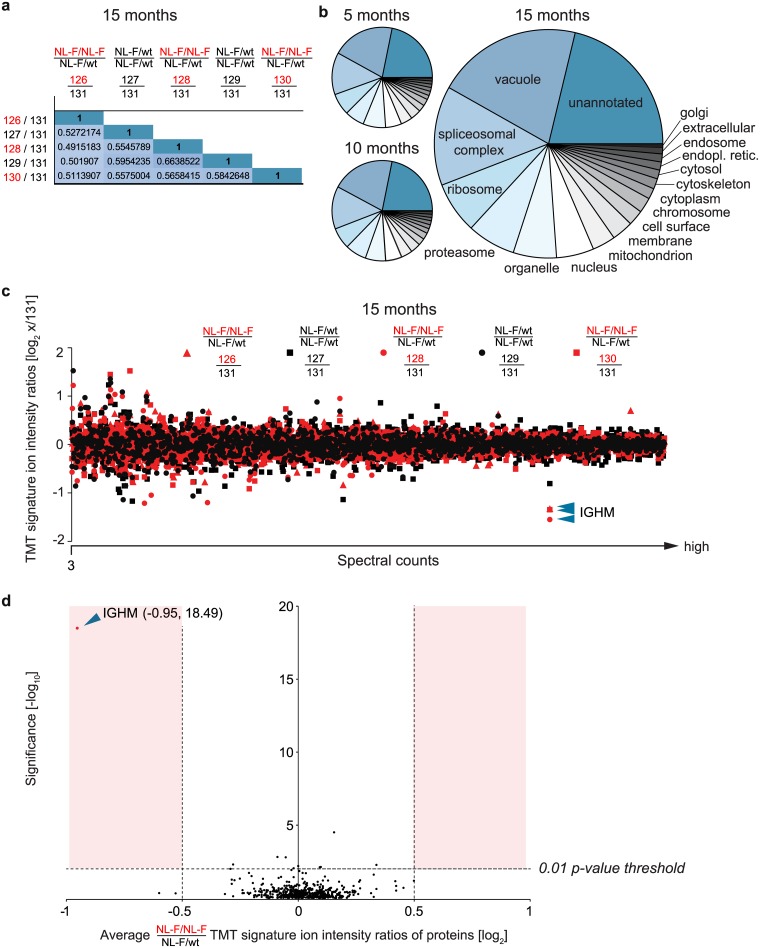
Global proteome datasets are characterized by consistent proteome coverage and a small number of proteins whose relative abundance ratios change in *APP*^*NL-F/NL-F*^ mice. (**a**) Pearson correlation coefficient analyses of abundance ratios of all proteins in a given sample whose relative quantitations could be based on the TMT signature ion intensity profiles from a minimum of three MS^3^ spectra. The coefficients were computed from the protein abundance levels in pairwise comparisons of samples, using the protein abundance levels in one of the *APP*^*NL-F/wt*^ negative control samples as a reference. Note that the Pearson correlation coefficients were similar (ranging from 0.49–0.66) regardless of whether the numerator in the calculation of relative abundance data originated from protein level data of *APP*^*NL-F/NL-F*^ *or APP*^*NL-F/wt*^ brain homogenates, indicating that the additional *APP*^*NL-F*^ allele present in homozygotes had no impact on the overall proteome that was discernible by this crude measure. (**b**) Broad and consistent representation of ‘Cellular Components’ Gene Ontology categories in the three global proteome datasets. (**c**) Graph depicting the relationship between spectral counts (the number of spectra recorded for a given protein) and the relative abundance ratios of proteins in datasets collected at the 15-month time-point. Even at 15 months the abundance levels of very few proteins are divergent between homozygous and heterozygous *APP*^*NL-F*^ mice. Each dot in this graph depicts the relative abundance ratio of a protein. Because only proteins that had been quantified on the basis of a minimum of 3 TMT signature ion intensity profiles were considered, the low-end cut-off on the x-axis has the value ‘3’ and amyloid beta is absent from the figure. Data points derived from the most abundant proteins (i.e., those that gave rise to a large number of MS spectra) are shown further to the right along the x-axis. Proteins, whose abundance levels differed most dramatically, are farthest away from the horizontal midline of the graph that bisects the y-axis at ‘0’ (note also the log_2_ scale of the ordinate). The graph narrows toward the right, indicating that improved relative quantitation statistics provided by the larger number of mass spectra per protein, reduced ‘noise’ in the data. As described in detail in the legend for panel ‘a’, the abundance levels of proteins observed in one of the *APP*^*NL-F/wt*^ negative control brain samples served as a reference. Red and black markers depict protein level abundance ratios computed with the numerator originating from protein abundance level data of *APP*^*NL-F/NL-F*^ *or APP*^*NL-F/wt*^ brain homogenates, respectively. (**d**) Volcano plot of quantified proteins from trypsin-digested brain homogenates of 15-month-old mice. The relative abundance of each protein is represented as the average Log_2_ ratio of all biological replicates from homozygotes (labelled with TMT reagents 126, 128 and 130) over the heterozygous biological replicate labelled with TMT reagent 131. Only unique peptides with reporter ion intensities of at least 10,000 counts were used for the quantification of each protein. Only proteins with three or more unique peptides and a protein confidence (-LogP) of 15 or more are shown. No normalization of the TMT ratios was performed. Significance was calculated using the PEAKSQ method [19]. Blue arrowheads in panel c and d point toward the IGHM protein.

**Fig 3:**
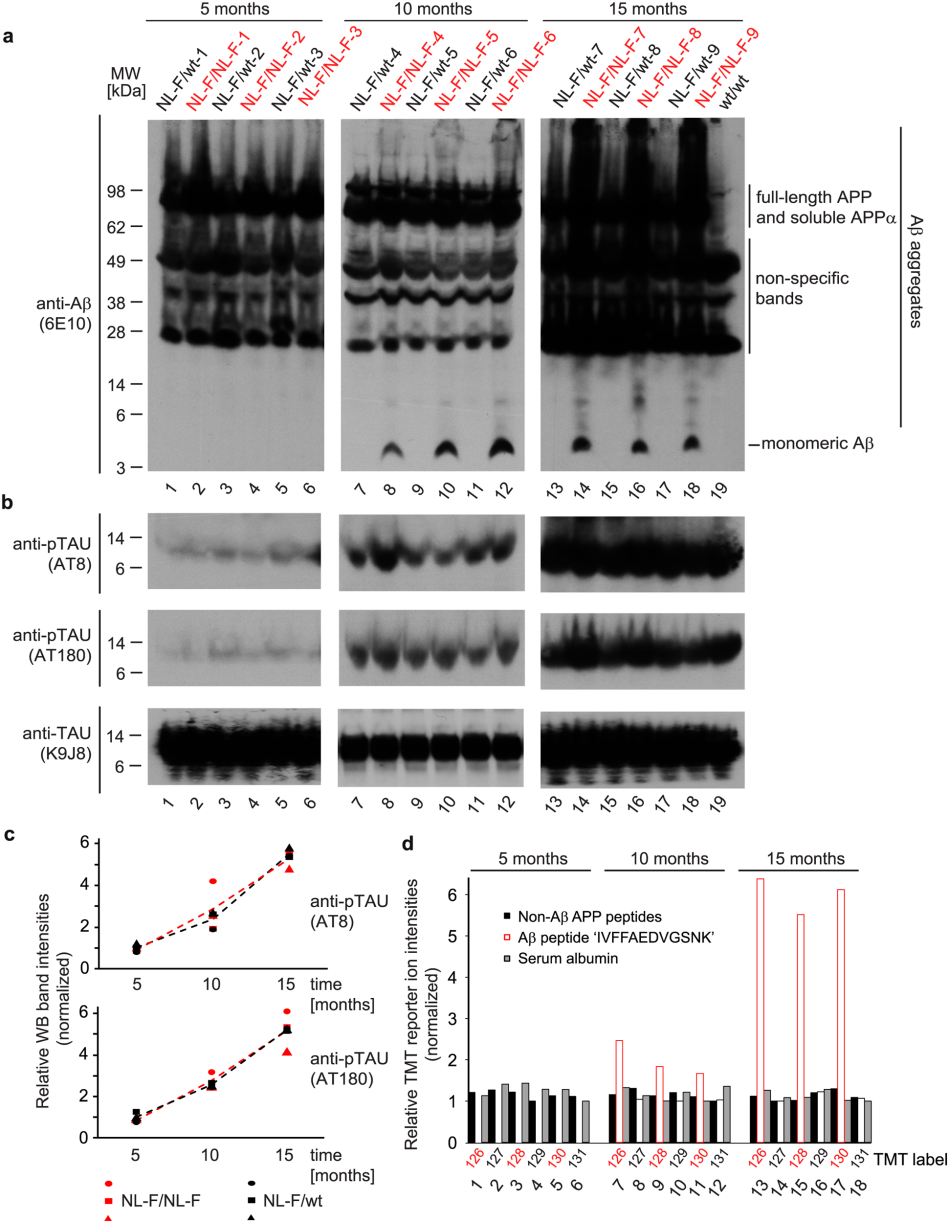
Profound age-dependent increase in Aβ levels in *APP*^*NL-F/NL-F*^ does not exacerbate age-related tau hyperphosphorylation relative to *APP*^*NL-F/wt*^ mice. (**a**) Western blot analysis of brain extracts from homozygous *APP*^*NL-F/NL-F*^ or heterozygous *APP*^*NL-F/wt*^ mice at the time points selected for global proteome analyses based on Aβ-directed 6E10 antibody. Note that signals migrating at the level of monomeric Aβ were not detected in 5-month-old mice, were visible at similar levels in brain extracts of 10- and 15-month-old *APP*^*NL-F/NL-F*^ mice but were not detected in *APP*^*NL-F/wt*^ brain extracts at any age tested. Notably, the extensive smearing of Aβ-reactive signals in samples derived from 15-month-old *APP*^*NL-F/NL-F*^ mice reflect the profound accumulation of SDS-resistant Aβ aggregates at that age. (**b**) Age-dependent, yet Aβ-independent, increases in phospho-occupancy levels of previously established tau phosphorylation sites in *APP*^*NL-F/NL-F*^ and *APP*^*NL-F/wt*^ mice. Western blots of brain extracts were probed with antibodies directed against total tau (K9JA), or the specific tau phosphorylation sites pS202/pT205 (AT8) and pT231 (AT180). (**c**) Relative quantitation of phospho-tau occupancy levels observed. (**d**) Relative TMT reporter ion intensities assigned to Aβ-specific peptides, non-Aβ APP-peptides or serum albumin-derived peptides in brain extracts of mice aged 5, 10 or 15 months. Note that confidence values for all peptide-to-spectrum matches considered exceeded a 0.05 false discovery rate cutoff threshold. For each age group, intensities of TMT reporter ions were normalized (see Materials and methods for details).

**Fig 4:**
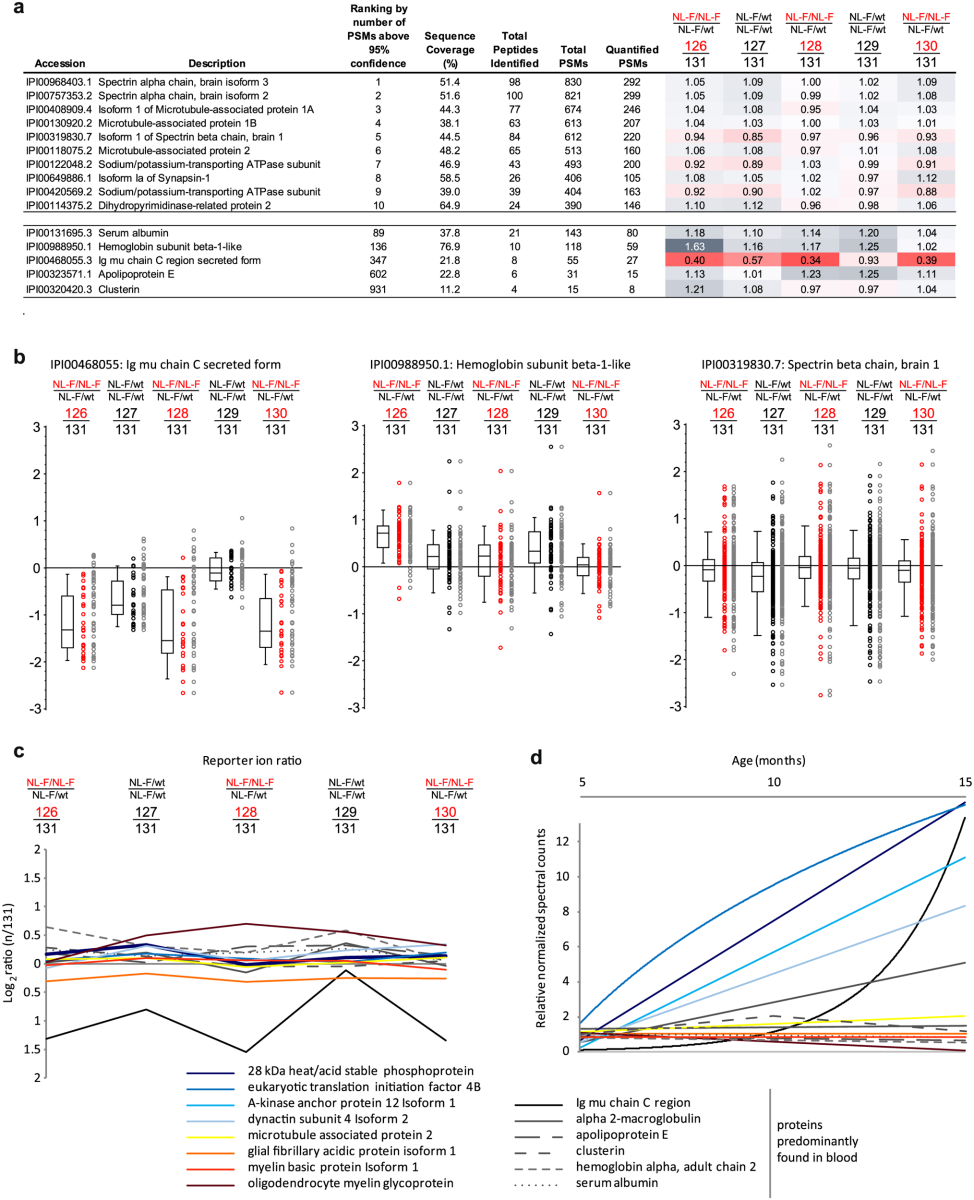
Selective depletion of IGHM but no other blood proteins in *APP*^*NL-F/NL-F*^ mice. (**a**) Subset of proteins identified and quantified by global proteome analyses in brain extracts of 15-month-old *APP*^*NL-F/NL-F*^ and *APP*^*NL-F/wt*^ mice (see S1 Table for details). The steady-state levels of all proteins identified on the basis of >3 peptide-to-spectrum matches were largely identical between *APP*^*NL-F/NL-F*^ and *APP*^*NL-F/wt*^ mice at this age. More specifically, median abundance ratios (computed with abundance levels of one *APP*^*NL-F/wt*^ biological replicate serving as the denominator for the calculation of ratios) fell within a narrow window of ratios spanning relative abundances of 0.7 to 1.3. The single exception to this consistent observation represented the IGHM protein, whose levels were more than two-fold reduced in *APP*^*NL-F/NL-F*^ mice, when compared to the respective levels in *APP*^*NL-F/wt*^ mice. Other blood proteins identified and quantified also deviated slightly from a 1:1 ratio for all comparisons but did not exhibit the same selective reduction in their steady-state levels as that observed for IGHM in *APP*^*NL-F/NL-F*^ mouse brain extracts. (**b**) Selected box plots depicting the relative abundance of peptides assigned to proteins that are known to reside primarily in blood. Black circles: relative abundance of peptides whose identification passed a false discovery rate threshold of 0.05. Red circles: relative abundance of peptides not considered for quantification because they were either identified more than once (accounting for a majority of eliminated quantitations) or did not pass the false discovery rate filter applied. Note the distinct ratios of peptides assigned to IGHM, a ratio profile that set it apart from other blood proteins (e.g., hemoglobin subunit beta-1) or abundant neuronal proteins (e.g., spectrin beta chain). (**c**) Direct comparison of relative abundance levels of IGHM, a subset of blood proteins and several other abundant proteins in brains of homozygous *APP*^*NL-F/NL-F*^ and heterozygous *APP*^*NL-F/wt*^ mice at 15 months. (**d**) Comparison of the relative abundance levels of proteins depicted in panel ‘**c**’ on the basis of spectral counts that underlie their identifications at the three mouse ages investigated in this study. The graph reveals a pronounced upregulation of IGHM levels in older mice. However, it also indicated that several other proteins, including eukaryotic translation initiation factor 4B, A-kinase anchor protein 12 and dynactin subunit 4, were also more than five-fold upregulated during the 15-month-time-course of the study, yet failed to exhibit a striking difference in their abundance ratios (compare with panel ‘**c**’).

The post-acquisition analysis of datasets followed a recently published workflow [15]. Briefly, mass spectra and tandem mass spectra were converted into polypeptide sequence information and MS^3^ reporter ion intensities were converted into quantitative data using Proteome Discoverer (Version 1.4, Thermo Fisher Scientific) and PEAKS Studio (Version 8, Bioinformatics Solutions Inc., Waterloo, ON, Canada). The two programs provided independent and complementary quantitative proteomic data sets. Polypeptide sequencing was carried out using the Mascot and Sequest HT algorithms in Proteome Discoverer and the *De Novo*, PEAKS DB, PEAKS PTM and SPIDER algorithms in PEAKS Studio. The international protein index (IPI) *Mus musculus* (Version 3.87) protein sequence database was used for spectral matching. For all algorithms employed, the precursor ion and product ion mass tolerances were set to 20 ppm and 0.4 Da, respectively, and the enzyme cleavage specificity was set to trypsin, with a maximum of two missed cleavages allowed. Pyridylethylation of cysteines and covalent modifications of primary amines with 6-plex TMT reagents were treated as fixed modifications. Oxidations at methionine, deamidations at asparagine or glutamine, and phosphorylation at serine, threonine or tyrosine were considered variable modifications. False discovery rate estimation in Proteome Discoverer was performed on the Percolator algorithm using the q-value for validation [16]. Gene Ontology (GO) analyses of ‘Cellular Component’ categories were undertaken with ProteinCenter software (Version 3.12; Thermo Fisher Scientific) and included all proteins from the three datasets, which had passed the aforementioned stringency filters. The graph depicting TMT signature ion intensity ratios as a function of spectral counts (Fig 2c) was generated in Excel following the import of filtered Proteome Discoverer datasets as tab delimited data files. Relative intensities of TMT reporter ions shown for APP-derived peptides (Fig 3d) were normalized to the intensity of the TMT reporter ion assigned to the least intense non-Aβ APP-derived peptide depicted. Differences in relative abundance ratios were emphasized by assigning background colors whose intensities corresponded to number values, with red and grey colors indicating ratios below and above 1, respectively. A subset of graphs depicting TMT ratios were exported from Proteome Discoverer (Fig 4b). Summary graphs depicting the TMT ratios (Fig 4c) and spectral counts (Fig 4d) of blood proteins and representative abundant brain proteins were generated in Excel by extracting TMT ratios or spectral counts from the master protein list of combined datasets (S1 Table). The mass spectrometry proteomics data have been deposited to the ProteomeXchange Consortium via the PRIDE [17] partner repository with the dataset identifier PXD004439.

### SDS-PAGE analyses and Western blot

All Western blot reagents, unless specified, were purchased from Thermo Fisher Scientific (Burlington, ON, Canada). Protein samples were mixed with LDS Sample Buffer (catalog number B0007) in the presence of 10% 2-mercaptoethanol and boiled at 70°C for 10 min before loading. Samples were analyzed by electrophoresis at 140 V in 4–12% Bis-Tris plus gels (catalog number NW04125BOX) in MES SDS running buffer (catalog number NP0002). The separated proteins were transferred from gels to polyvinylidene fluoride (PVDF) membranes for 90 min at 45 V. Subsequently, Western blot membranes were blocked for 2 h in tris-buffered saline with 0.1% Tween 20 (TBST) containing 5% fat-free milk and probed overnight with the respective primary antibodies. Next, Western blot membranes were incubated with horseradish peroxidase-conjugated anti-mouse or anti-rabbit secondary antibodies (Bio-Rad Laboratories, Inc., Hercules, CA, USA) for 2 h. Protein band signals were visualized by incubation with ECL reagent (catalog number 4500875; GE Health Care Canada, Inc., Mississauga, ON, Canada), followed by exposure to blue Bioflex X-ray films (Clonex, Markham, ON, Canada). The density of bands was measured using ImageJ software (National Institutes of Health, Bethesda, MD, USA) [18]. Where indicated, actin served as a loading control; Coomassie staining was performed on blot membranes to confirm equal loading in certain cases.

### ELISA-based quantitation of total IgM and IgG levels, as well as levels of phosphorylcholine-directed IgMs

Levels of IgM (catalog number 88–50470) and IgG (catalog number 88–50400) in mouse serum samples were measured with mouse Ready-Set-Go ELISA kits (eBioscience Inc., San Diego, CA, USA) following the manufacturer’s protocol. The IgM and IgG concentration were calculated by fitting measured values to the standard curve using four-parameter logistic regression. The anti-Phosphorylcholine IgM levels in mouse serum samples were measured with anti-phosphorylcholine IgM ELISA kit (catalog number 680-110-PCM; Alpha Diagnostic Intl Inc., USA).

### Statistical analyses

**Fig 5:**
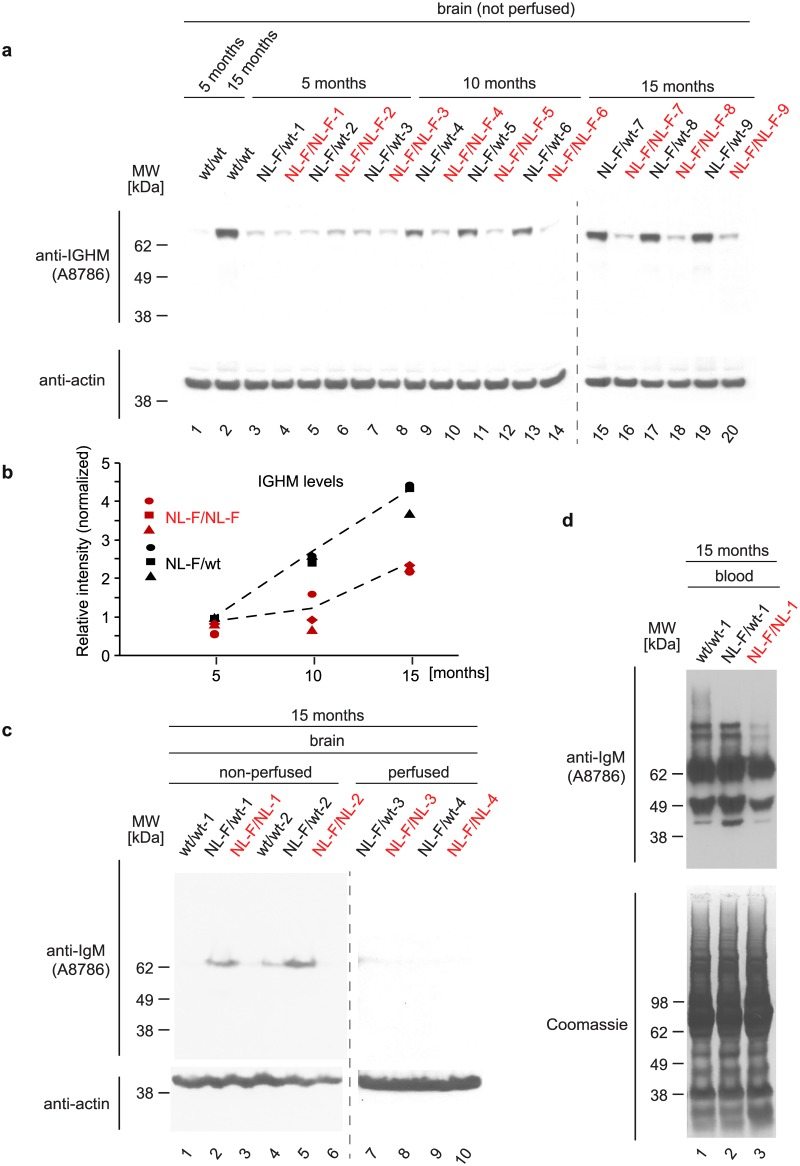
Gradual accumulation of Aβ in *APP*^*NL-F/NL-F*^ mice causes IGHM depletion in the blood, not the brain. (**a**) Validation of selective depletion of IGHM steady state levels in 10 and 15 month old *APP*^*NL-F/NL-F*^ mice relative to *APP*^*NL-F/wt*^ mice. Brain samples were adjusted for equal protein concentrations based on BCA assay. Actin served as a loading control in these experiments. Note the general increase in IGHM levels over the 15 month course of the experiment observed in *APP*^*WT/WT*^ and heterozygous *APP*^*NL-F/wt*^ mice. (**b**) Licor-based relative quantitation of Western blot signals of IGHM levels shown in panel ‘a’. (**c**) IGHM levels in perfused brains were negligible. Western blot-based comparison of IGHM levels in non-perfused and perfused brain tissue of 15-month-old *APP*^*WT/WT*^, *APP*^*NL-F/NL-F*^ and *APP*^*NL-F/wt*^ mice. (**d**) The reduction in IGHM levels in *APP*^*NL-F/NL-F*^ mice manifests in the blood, indicative of a systemic reduction of this protein.

**Fig 6:**
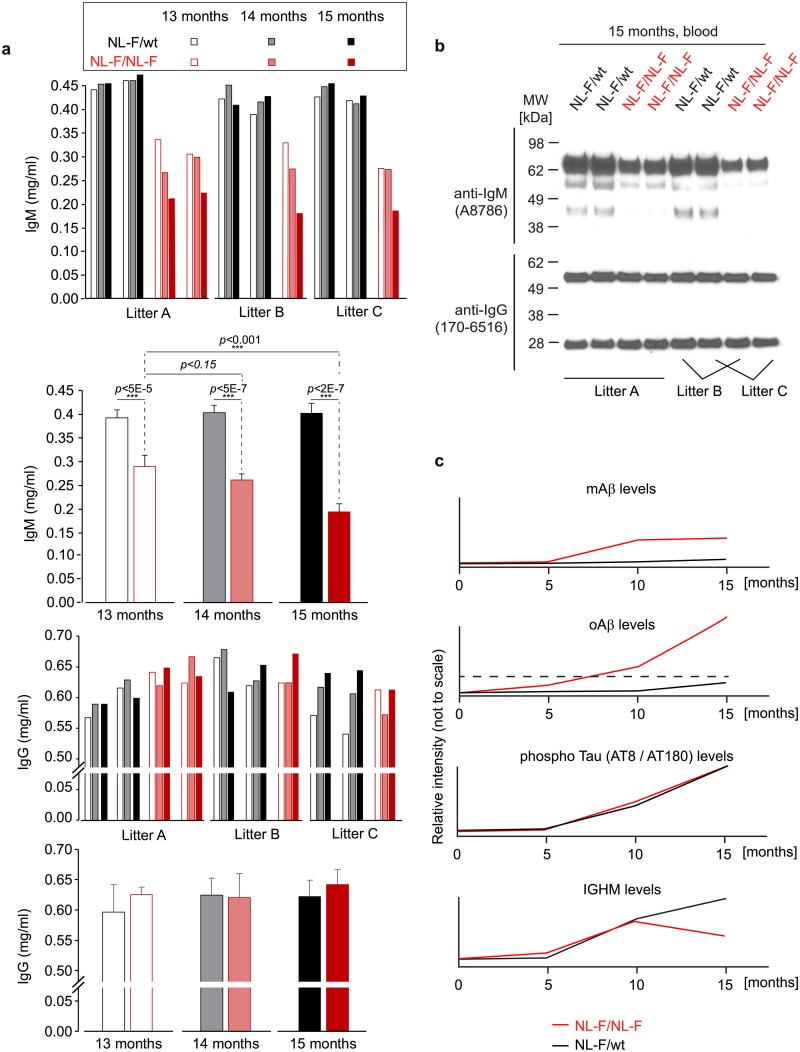
Depletion of IGHM in the blood is progressive and does not extend to immunoglobulin G. (**a**) Quantitative ELISA analyses of serum samples harvested from three mouse litters at three different time-points (i.e., at ages of 13, 14 and 15 months) established that the relative depletion of IGHM levels in *APP*^*NL-F/NL-F*^ mice progressively increases in the time window investigated, and does not extend to immunoglobulin G (IgG) levels. Whenever *p*-values computed for relative ELISA signals passed significance thresholds, this was indicated in the graph, with triple (***) and single (*) asterisks designating *p*-values < 0.001 and <0.5, respectively. (**b**) Western blot analyses of a subset of serum samples utilized in panel ‘a’ further corroborated robust depletion of IGHM levels, but also revealed that IgG levels were not similarly depleted in direct comparisons of heterozygous *APP*^*NL-F/wt*^ and homozygous *APP*^*NL-F/NL-F*^ mice. (**c**) Schematic graphs summarizing the key trends in relative protein abundances observed in this study (not to scale).

For quantifications in Proteome Discoverer, all peptides scoring above the 0.05 confidence threshold were incorporated into protein quantifications, whereas PEAKS Studio allowed only unique peptides to be used for protein quantification. For TMT analyses, one of the reporter ion channels was randomly selected from each age group to serve as a common comparator (denominator), allowing the assessment of variation among biological replicates of both the heterozygous and homozygous groups. Median reporter ion ratios and quantiles for quantified proteins were determined in Proteome Discoverer. Pearson correlation coefficients of reporter ion ratios (Fig 2a) were calculated in Microsoft Excel. Because the statistical reliability of relative quantitations for a given protein increases with the number of TMT signature ion profiles recorded for its peptides, relative quantitations were dismissed unless they were based on three or more distinct TMT signature ion spectra. Hierarchical clustering analyses and the calculation of fold changes and significance were conducted with the PEAKS Q function of PEAKS Studio, For volcano plot analyses, significance was calculated using the PEAKS Q method [19]. For the comparison between the two genotypes, statistical analysis was performed by paired (Figs 3c and 5b) or unpaired (Fig 6a) Student *t* test. The comparison of proportions was based on a one sample *t* test. In all cases, *p* < 0.05 was considered significant. Where included, results from these analyses depict data variability as mean (SD).

## Results

### Design of quantitative analysis to capture Aβ1-42-dependent changes to proteomes

To detect changes to steady-state protein levels in response to Aβ1–42 release, the study made use of the *APP*^*NL-F*^ model [12] (Fig 1a). As negative controls heterozygote *APP*^*NL-F/wt*^ mice, which were known to exhibit a profoundly reduced pathology relative to *APP*^*NL-F/NL-F*^ homozygotes were used (Fig 1b). First signs of increased Aβ1–42 production in *APP*^*NL-F/NL-F*^ mice can be observed at around six months of age. At 12 months of age, the mice increasingly exhibit Aβ1–42 plaque deposition, and 18-month-old *APP*^*NL-F/NL-F*^ mice had been described to present with memory impairments [12]. Here, cohorts of three homozygous and heterozygous mice each were sacrificed at 5, 10 and 15 months of age to capture proteome shifts before plaque deposition has occurred or when early or mid-stage effects of Aβ1–42 overproduction manifest in the mice (Fig 1c).

To minimize the occurrence of inadvertent post-translational modifications of proteins during sample handling, mouse brains were rapidly dissected from deeply anaesthetized mice and subjected to homogenization by bead beating in the presence of sodium dodecyl sulfate (SDS) that had been pre-heated to 95°C. To eliminate run-to-run variances as confounders in downstream relative comparisons of peptide mixtures, primary amines present at the N-termini and lysine side-chains of peptides were labeled with isobaric TMT [20]. Subsequently, biological replicates and controls were pooled and co-analyzed by deep global proteome analyses on an Orbitrap Fusion Tribrid instrument. The machine was configured to first undertake survey scans in its orbitrap mass analyzer, then acquire product ion spectra from the most intense precursor ions that carried at least two positive charges in the linear ion trap, and to, finally, collect TMT signature ion profiles in the orbitrap following high-energy collision-induced dissociation of the ten most intense product ion peaks (Fig 1d).

The post-acquisition analysis of datasets made use of Percolator, a semi-supervised learning algorithm that filters high quality spectra from shotgun proteomics datasets [16]. The subsequent assignment of spectra to peptide sequences in the international protein index (IPI) mouse database was based on Mascot and Sequest matching algorithms. The study design allowed the direct comparison of steady-state abundance ratios of more than 2,500 proteins identified in homozygous *APP*^*NL-F/NL-F*^ and heterozygous *APP*^*NL-F/wt*^ mouse brains using a six-plex co-analysis format. It further provided the ability to compare the abundance levels of a given protein across the three ages based on the spectral counting method (see below for details on this aspect of the methodology) [21] (Fig 1e).

### High reproducibility of global proteome datasets and low divergence of proteomes from homozygous and heterozygous *APP*^*NL-F*^ mice

When combined, the global proteome analyses led to the identification of 2,526 proteins, including 12 proteins encoded by well-confirmed AD loci (*APP*, *APOE*, *PSEN2*, *CLU*, *PICALM*, *BIN1*, *PTK2B*, *RIN3*, *ZCWPW1*, *FERMT2*, *ABI3*, and *TREM2*). All these proteins were identified and quantified on the basis of more than three unique PSMs and TMT signature ion distributions in at least one of the three datasets. Of these, 1,761 proteins were observed with >20% sequence coverage (see S1 Table). To gauge the reproducibility of the analysis method, we calculated the Pearson correlation coefficient of protein abundance ratios across biological replicates of 15-month-old homozygous or heterozygous mice. Based on this analysis, pair-wise comparisons of abundance ratios gave rise to Pearson coefficients between 0.49 and 0.68, indicating considerable correlation of abundance ratios across biological replicates. Of note, Pearson correlation coefficients computed for direct comparisons of samples derived from homozygotes and heterozygotes did not differ in this regard, indicating that the presence of the Aβ1–42 induced pathology exerted no effect on the overall proteome that was discernible by this crude measure (Fig 2a). We next compared the extent and reproducibility to which the global proteome was covered in the deep proteome analysis at the three different mouse ages. This led us to notice that the spectral counts, which supported a subset of protein identifications differed considerably across the three ages. However, whereas this proteome shift affected the levels of this subset of proteins individually, a GO analysis documented broad coverage of all cellular components, and more importantly, indicated that the assignment to GO terms was essentially identical in the global proteome datasets collected from samples at the three ages (Fig 2b). Second, we investigated the distribution of protein abundance ratios and determined to what extent these ratios depended on the number of TMT signature ion profiles reported for a given protein (Fig 2c). This analysis revealed that, with few exceptions (see detailed information in subsequent sections), the relative abundance ratios of all proteins was close to 0 (in Log_2_ space) and were more consistent from protein to protein (indicated by a narrower distribution), whenever quantitations were based on a larger number of TMT signature ion intensity profiles. The latter observation was expected and indicates that the influence of ‘noise’ (e.g., in the form of co-isolating peptides that can distort the intensity of individual TMT signature ion intensity profiles) diminishes as the statistics of peptide-based signature ion profiles underlying protein quantitations becomes increasingly robust. This distribution further indicated that the elevated production of Aβ1–42 in homozygotes had no pronounced effect on the overall proteome even in 15-month-old mice, despite their previously established pathology and plaque formation [12]. The same overall conclusion was also obtained when relative protein abundance levels of 15-month-old *APP*^*NL-F/NL-F*^ homozygotes and *APP*^*NL-F/wt*^ heterozygotes were graphed in volcano plot format (Fig 2d).

### Age-dependent increase in Aβ levels in *APP*^*NL-F/NL-F*^ mice does not exacerbate age-related tau hyperphosphorylation relative to *APP*^*NL-F/wt*^ mice

To explore the relative levels of Aβ1–42 production in *APP*^*NL-F/NL-F*^ homozygotes and *APP*^*NL-F/wt*^ heterozygotes at the three ages investigated, we next undertook Western blot analyses of the brain extracts, which had provided the biological material for the global proteome analyses. To facilitate direct comparisons of the relative abundances of signals recognized by the 6E10 antibody for all APP expression products concomitantly, signals for the low molecular mass monomeric Aβ peptide and higher molecular mass APP expression products, including the secreted APP cleavage products, were monitored on the same Western blot panel. This analysis revealed that a 6E10 antibody-reactive Western blot signal, whose migration is consistent with the presence of monomeric Aβ1–42, could only be observed in brain extracts from 10- and 15-month-old homozygotes (Fig 3a). Consistent with the presence SDS-resistant Aβ1–42 aggregates in homozygotes, closer inspection of the same Western blots, also revealed the presence of a 6E10 antibody-reactive smear that migrated in the denaturing SDS-PAGE gel with apparent molecular masses spanning from 4 to 250 kDa (the upper resolving limit of the gel). This smear was only weakly visible in 10-month-old homozygotes but became pronounced at 15 months of age. We next investigated if the elevated production of Aβ1–42 led to a reliable induction of tau phosphorylation at phospho-acceptor sites whose occupancy is known to be increased in preparations of hyperphosphorylated tau from postmortem AD brains (Fig 3b). Although there was a strong age-dependent increase in signals detected with the respective phospho-specific antibodies (relative to signals detected with an antibody reactive toward total tau) in 10- and 15-month-old mice, this increase did not correlate with NL-F allele status (Fig 3c). Although several peptides had been assigned to tau in the global proteome analyses, sequence coverage for this protein was not sufficient to assess its phosphorylation status by this orthogonal methodology. However, the increased production of Aβ1–42 seen in the Western blot analysis was readily detected also in the global proteome datasets. More specifically, the Aβ1-42-derived tryptic peptide with the (one letter code) amino acid sequence ‘IVFFAEDVGSNK’ was detected in brain extracts of 10- and 15-month-old *APP*^*NL-F/NL-F*^ homozygotes but not in *APP*^*NL-F/wt*^ heterozygotes, with levels at 15 months exceeding 10-month-levels of this peptide approximately three-fold (Fig 3d). Consistent with the interpretation that the elevated expression of Aβ1–42 in this AD mouse model does not influence steady-state levels of other APP-expression products, peptides assigned to APP that map to regions outside of the Aβ1–42 domain were present at identical levels in homozygotes and heterozygotes.

### Selective depletion of IGHM but no other blood proteins in *APP*^*NL-F/NL-F*^ mice

The close comparison of mass spectrometry datasets generated with brain homogenates from 5-month-old *APP*^*NL-F/NL-F*^ homozygotes and *APP*^*NL-F/wt*^ heterozygotes revealed no proteins whose steady-state levels were reproducibly changed. At 10 months of age, the most abundant proteins were again observed to be present at identical levels (Fig 4a). However, there was a small number of proteins, including serum albumin and hemoglobin subunits, whose levels deviated slightly in at least two of the homozygotes. The identity of these proteins revealed them to be blood-derived. We ascribed this observation to the fact that we had neither perfused the mice prior to the rapid dissection of their brains nor had rinsed the dissected brains externally with PBS, and therefore had perhaps carried slightly different amounts of blood through the sample preparation steps. Nevertheless, the presence of these blood proteins should not be considered a ‘contamination’ of the brain samples but rather reflects the fact that the functioning brain is intimately supported by an intricate network of blood vessels. Of note, whereas the aforementioned blood proteins shared a consistent abundance level ratio, the IGHM protein, which may also have originated in the blood, exhibited the most pronounced reduction in its levels in brains from 10-month-old *APP*^*NL-F/NL-F*^ mice, relative to its levels in brains from heterozygotes of the same age. Strikingly, whereas in brain extracts from 15-month-old mice the abundance levels of other blood proteins no longer stood out, levels of IGHM had further dropped in *APP*^*NL-F/NL-F*^ mice in all three biological replicates. This distribution, which was determined on the basis of 27 separately quantified PSM, was unique for IGHM and was independently observed by both the PEAKS and Proteome Discoverer algorithms, distinguishing it with a highly significant *p*-value from all other proteins in the volcano plot (blue arrowhead, Fig 2d) and in box plots summarizing TMT ratios of other robustly quantified proteins (Fig 4b).

We next compared the median TMT-based protein ratios observed for IGHM and several blood proteins, as well as highly abundant proteins, including proteins known to be associated with different cell types (i.e., glia, oligodendrocytes or neurons). This analysis further validated the unique depletion of IGHM in brain homogenates of *APP*^*NL-F/NL-F*^ mice, relative to the levels of this protein in *APP*^*NL-F/wt*^ littermates (Fig 4c). To learn whether levels of IGHM had changed relative to other proteins (independent of the NL-F allele status) at the three ages studied, we next made use of the spectral counting method. This approach exploits the observation that in samples of comparable complexity (analyzed under identical conditions) the numbers of mass spectra assigned to a given protein tend to correlate directly with its relative abundance levels. Most often spectral counting is used as an alternative to isobaric labeling techniques. Because in this study *APP*^*NL-F/wt*^*- and APP*^*NL-F/NL-F*^-derived samples had to be mixed for TMT analyses, here the spectral counting method was not used to explore how the allele status affected the levels of a given protein but informed about general trends of a protein’s abundance at the three ages investigated (Fig 4d). In other words, we compared the number of spectra assigned to IGHM or other proteins at the three different ages covered in the time-course analysis in all biological replicates combined. Whereas Western blot analyses would have limited the number of proteins we could survey, this approach allowed us to compare the abundance profiles of IGHM to those of a large number of other proteins. According to this analysis, and in contrast to other blood proteins, IGHM levels had indeed increased more than ten-fold when comparisons were undertaken between 5- and 15-month-old *APP*^*NL-F*^ mice. However, IGHM levels were not unique in this regard, as levels of several other proteins, including the eukaryotic translation initiation factor 4B, A-kinase anchor protein 12, dynactin subunit 4 and the 28 kDa heat/acid stable phosphoprotein, had similarly increased during this time-span. Critically however, unlike IGHM, the latter proteins did not exhibit changes in their relative amounts in brain extracts from 15-month-old *APP*^*NL-F/NL-F*^ *and APP*^*NL-F/wt*^ littermates (Fig 4c), suggesting that the elevated levels of Aβ1–42 in *APP*^*NL-F/NL-F*^ mouse brains had not resulted in an overall slowing of the cellular production of proteins whose levels are profoundly increased in 15-month-old mice. Notably, we also did not observe significant changes among robustly quantified proteins encoded by well-confirmed AD loci. Taken together, these observations identified IGHM as a candidate protein, whose levels were selectively diminished in *APP*^*NL-F/NL-F*^ homozygotes.

### Gradual accumulation of Aβ in *APP*^*NL-F/NL-F*^ mice correlates with IGHM depletion in the blood, not the brain

To validate the decreased levels of IGHM in *APP*^*NL-F/NL-F*^ mice, revealed by global mass spectrometry analyses, and more broadly investigate expression levels of this protein in 5-, 10- and 15-month-old mice, Western blot analyses of brain homogenates from *APP*^*NL-F/NL-F*^ and *APP*^*NL-F/wt*^ mice were conducted (Fig 5a). These analyses confirmed the relative reduction of steady-state IGHM levels in *APP*^*NL-F/NL-F*^ homozygotes (Fig 5b) but also uncovered that levels of this protein are barely detectable at 5 months of age, and increase with age in 10- and 15-month-old mice. To clarify if the relative reduction of IGHM levels documented in *APP*^*NL-F/NL-F*^ homozygotes was the consequence of a systemic reduction of IGHM or occurred locally in the brain, the Western blot analyses were repeated with brain samples from which blood had been drained by perfusion with phosphate buffered saline prior to their dissection (Fig 5c) and blood samples collected from 15-month-old *APP*^*NL-F/NL-F*^ homozygous and *APP*^*NL-F/wt*^ heterozygous mice (Fig 5d). This experiment gave rise to faint IGHM antibody reactive bands in perfused brains, thereby documenting that this protein is virtually absent within the mouse brain. However, this analysis also revealed pronounced IGHM levels in the blood of *APP*^*NL-F/wt*^ heterozygotes and lower levels of this protein in the blood of *APP*^*NL-F/NL-F*^ homozygotes. An expanded screen of a larger number of samples from our colony of *APP*^*NL-F*^ mice revealed considerable variability in IGHM serum levels in mice younger than 15 months (S1 Fig) that was not anticipated based on the robust reduction of IGHM-levels in the global proteome and Western blot analyses of brain samples (Fig 5a). A similar extended screen of serum samples from *APP*^*NL-F*^ mice that were 15 months of age, mostly confirmed the previously observed reduction in IGHM levels but also revealed that there is additional variability in the levels of this protein across litters in mice of the same age (S2 Fig). The latter analysis also revealed two outliers, in which IGHM-levels were inverted relative to other pairings and also were conspicuous in that the two mouse pairs exhibited an unusual amount of hair loss. At the time of writing, a total of 16 pairs of 15-month-old homozygous *APP*^*NL-F/NL-F*^ and heterozygous *APP*^*NL-F/wt*^ mice have been analyzed, and in 14 of these pairs levels of IGHM were observed to be lower in the homozygotes, relative to their heterozygote littermates (*p* = 0.0004, based on *t* test between proportions). Thus, taken together, these experiments confirmed the differences in IGHM levels in homozygous and heterozygous *APP*^*NL-F*^ mouse brains from 15-month-old mice but also drew attention to the considerable variability in the levels of this protein, and indicated that the relative depletion of steady-state IGHM levels most likely manifests systemically in the blood.

### Depletion of IGHM in the blood of *APP*^*NL-F/NL-F*^ mice reflects selective loss of IgMs that does not extend to IgGs

We next sought to investigate the apparent depletion of IGHM in the blood of *APP*^*NL-F/NL-F*^ mice in a way that minimizes potential confounders inherent to the variability across litters. We also were interested in determining if the relative depletion extends to the main IgG class of immunoglobulins. To this end, we identified three litters of heterozygote intercrosses of *APP*^*NL-F/wt*^ mice that each comprised same sex (females) *APP*^*NL-F/wt*^ and *APP*^*NL-F/NL-F*^ littermates. At the ages of 13, 14 and 15 months, a small amount of serum was collected in order to quantify IgM and IgG levels using a commercial ELISA format (Fig 6a). This experiment revealed that IgM levels remained stable in heterozygous *APP*^*NL-F/wt*^ mice within the age window analyzed but also revealed a significant, approximately 50% reduction of this immunoglobulin class in homozygous *APP*^*NL-F/NL-F*^ mice. Moreover, a closer evaluation of IgM levels in 13-, 14- and 15-month-old mice established a significant month-to-month reduction in IgM levels in this cohort. A similar relative reduction was not observed for IgG immunoglobulins in direct comparisons of *APP*^*NL-F/wt*^ and *APP*^*NL-F/NL-F*^ littermates of the same age, or in consecutive serum samples from mice in this cohort at the ages of 13, 14 and 15 months. Western blot analyses of a subset of serum samples from each of the three litters validated these results by confirming that the lower ELISA IgM signals in serum from *APP*^*NL-F/NL-F*^ reflected the lower IGHM serum levels, and also corroborated the steady levels of IgG heavy and light chains across *APP* allele status and ages tested (Fig 6b).

### Depletion of IGHM is not observed in 12-month-old CRND8 mice, despite their high levels of Aβ production

Does the selective IGHM reduction extend to another mouse model known to produce excessive amounts of Aβ? To address this question, we capitalized on local access to CRND8 mice which express highly elevated levels of the human APP transgene carrying the Swedish and Indiana mutations as well as coding for a *PSEN1* transgene exhibiting two familial AD mutations [22]. Because CRND8 mice express considerably higher levels of Aβ already at a younger age than APP^*NL-F-NL-F*^ mice, these experiments were undertaken with 12-month-old mice, obtained by intercrossing wild-type mice with CRND8 mice that carry several copies of the transgenes within an array that has been heterozygously inserted on one chromosome. The transgene status of the littermates was determined by PCR of genomic DNA and Aβ-directed immunoblotting of brain extracts. Subsequently, serum samples collected from the mice were analyzed by Western blotting with antibodies directed against IgM and IgG. As expected, the overexpression of the transgene array caused not only the strong appearance of Western blot signals that can be attributed to monomeric Aβ but also revealed intense high molecular mass bands, reflecting the overproduction of the APP precursor gene. Intriguingly, despite the pronounced production of Aβ in transgenic animals, these mice did not exhibit selective loss of IgM levels at the age of 12 months. Instead, the results from this analysis indicated an increase in IgG levels in transgenic mice relative to their wild-type littermates that was most strikingly seen in a litter of four mice (S3 Fig, **Panel b, Litter D**).

## Discussion

The current study set out to identify changes to the proteome in response to elevated Aβ1–42 production. To our knowledge, this was the first investigation of its kind in an advanced mouse model of AD-like Aβ1–42 amyloidosis. The study made use of sophisticated technology and workflows to quantify the relative abundance of proteins in a six-plex format at three mouse ages. It documented that chronically elevated Aβ1–42 production had no discernible effect on steady-state protein levels of the >2,500 most abundant brain proteins and yet was associated with a selective and pronounced systemic reduction of IGHM protein levels in the blood of homozygous *APP*^*NL-F/NL-F*^ mice, relative to heterozygous *APP*^*NL-F/wt*^ mice (Fig 6c). Whereas a selective depletion of Aβ1-42-specific antibodies might be expected, a relative reduction of total IGHM levels was not anticipated. Importantly, this molecular phenotype was observed in littermates, which were generated by crossing heterozygous knock-in mice, excluding genomic drift as an explanation. Moreover, littermates were most often housed in the same cage and were exposed to the same food, thereby minimizing the chance that exposure to different sets of antigens could have caused a selective immune deficiency only in *APP*^*NL-F/NL-F*^ homozygotes.

A recent mass spectrometry study, which also incorporated isobaric labeling in its workflow, reported on early changes to the global blood proteome in individuals that presented with mild cognitive impairment (MCI) or AD [23]. Because the study was based on human blood samples and had to rely on complex clean-up steps for the removal of the six most abundant plasma proteins, it recorded considerable sample-to-sample differences. Nonetheless, it concluded that thirty out of approximately a hundred proteins detected were significantly altered. Notably, IGHM was one of these proteins, but in contrast to the AD mouse model data presented here, its levels were described to be upregulated in MCI and AD cases relative to controls. In addition to this recent large-scale study, there have been several reports which documented altered IgM levels in individuals diagnosed with MCI or AD (see below). However, none of these prior studies settled the question, if changes to IgM levels were merely correlative, represented a risk factor for acquiring the disease, or were caused by it. Data presented in this study are indicative of a causative relationship between the chronically elevated production of Aβ1–42 and the relative depletion of IGHMs.

A surprising observation in this study, owed to the fact that mouse brains were not perfused during their preparation, has been the bulk reduction in IgM levels, which could indicate increased immunoglobulin class switching or reflect the manifestation of a rare form of dysgammaglobulinemia, known as selective IgM deficiency (SIgMD). There are several known causes, which may underlie SIgMD, including excessive induction of regulatory T cells, formerly known as IgM isospecific suppressor T cells, an immune cell population tasked with protecting against autoimmune diseases [24]. It has long been understood that the immune system employs an intricate antibody selection and maturation program to minimize the occurrence of adversary self-directed antibodies, which may lead to auto-immune diseases. Because natural IgMs represent the first line of the humoral immune defense, they often exhibit relative weak affinity and somewhat promiscuous binding toward a wide variety of antigens. However, it has been shown that a considerable proportion of IgMs evolved to serve specific purposes. For example, IgMs binding to phosphorylcholine neo-epitopes on oxidized low-density lipoprotein (oxLDL) are surprisingly abundant. It has been speculated that this is in part a result of the fact that these antibodies also bind to apoptotic cells [25] to facilitate their efferocytosis (removal of dead cells) [26]. Thus, a subpool of IgMs appear to serve a specific role that was acquired as an evolutionary adaptation to programmed cell death and is preserved in a range of mammalian species, including humans [27]. The critical role these antibodies play for the organism can be deduced from the observation that approximately 30% of natural antibodies bind to oxidation-specific neoepitopes [25]. Current attempts by this team to clarify if this pool of anti-phosphorylcholine IgMs is affected in *APP*^*NL-F/NL-F*^ homozygous mice have remained inconclusive (S4 Fig). However, support for the notion that Aβ1–42 might bind to and exhaust this pool of IgMs emerged in an earlier study [28]. More specifically, the authors documented that IgMs directed against phosphorylcholine were lower in serum samples from patients with dementia, and the likelihood of dementia or AD was twice as high for individuals with the lowest levels of this IgM subpool. Due to the study design, no conclusion could be drawn if these low levels predisposed individuals to develop AD or were the consequence of it [28]. However, a more recent study that quantified anti-phosphorylcholine antibody titers in human plasma samples from AD and control cases disputed these findings [29]. Interestingly, there also is a strong inverse correlation between APOE and oxLDL levels. ApoE-deficient mice are characterized by high levels of oxLDL, and the APOE-ɛ4 allele is not just a risk factor for AD but also for atherosclerosis, a disease characterized by depletion of the specific subtype of IgM recognizing oxLDL. Follow-up investigations are needed to shed light on the specific IgM subtype affected.

One of the observations made in this study was that monomeric Aβ1–42 signals escaped detection in Western blot analyses of brain extracts from *APP*^*NL-F/wt*^ heterozygotes, even when derived from 15-month-old mice. In contrast, pronounced signals of monomeric Aβ1–42 and its aggregated derivatives were detected in 10- and 15-month-old *APP*^*NL-F/NL-F*^ homozygotes, consistent with the interpretation that efficient clearance mechanisms must exist. The latter keep levels of Aβ1–42 in check, so long as the rate of its production remains low but appear to get overwhelmed in this mouse model once Aβ1–42 generation is coded by two NL-F knock-in alleles. Several mechanisms have been shown to exist that may contribute to this clearance. Data from the current study further add to a body of literature that suggests antibody-based clearance may play a critical role in the brains ability to cope with Aβ1–42 formation. Consistent with this interpretation, IGHM levels were observed to be elevated in *APP*^*NL-F/wt*^ heterozygotes relative to wild-type littermates, but were most often reduced in homozygous *APP*^*NL-F/NL-F*^ mice. Evidence for the existence of Aβ-directed auto-antibodies in B-cell lines established from individuals with a clinical diagnosis of AD were first reported more than 20 years ago [30]. Since then, several studies have reported on the existence of Aβ-reactive auto-antibodies and evaluated their diagnostic merit [31]. While these antibodies may play a physiological role, they also pose a challenge for the development of blood-based diagnostics, i.e., unless efforts are undertaken to promote the dissociation of pre-existing Aβ-immunoglobulin complexes [32], their existence may add to variability in detection results by masking the true levels of Aβ in the blood [33]. Whereas most previous studies did not specifically investigate interactions between Aβ and IgM, one study documented the existence of IgM-Aβ complexes in blood but did not detect differences between MCI and AD samples [34]. However, the same authors subsequently reported a reduction of IgM auto-antibodies against pyro-glu Aβ [35]. Others have shown that there is a correlation between CSF and plasma levels of Aβ in healthy individuals but not in MCI or AD cases [36]. Could it be that IgMs selectively deplete oligomeric Aβ in the blood of patients? Adding fuel to this concept was a study, which reported that auto-antibodies against oligomeric Aβ, but not monomeric Aβ, were selectively depleted in AD [37]. More importantly, age of onset was significantly correlated with auto-antibody levels against oligomeric Aβ, i.e., high levels of auto-antibodies were proposed to protect against the disease. A recent study capitalized on this concept by generating proof-of-principle data which exploited a specific IgM for the capture of oligomeric (over monomeric) Aβ [38].

An alternative explanation for the reduction of IgM levels is the possibility of immunoglobulin class switching. A candidate mediator of the underlying biology that should be considered in this context is the Toll-like receptor 4 (TLR4), which has been shown to mediate T-cell independent class switching [39]. Its potential relationship to the biology investigated here stems from the fact that TLR4 has independently been suggested to play a role in Aβ uptake [40], and its levels were reported to be increased on peripheral blood mononuclear cells from individuals with AD [41]. Consistent with such a scenario, Aβ has already been shown to activate signaling downstream of TLR4 [42]. In the current report, we did not observe evidence of altered IgG levels in the *APP*^*NL-F*^ mouse model but our tentative results generated with the CRND8 model indicated that levels of IgG might be increased relative to wild-type littermates in mice expressing the transgene array. Further investigations of Aβ, beyond the scope of this study, may provide explanations for why the two Aβ amyloidosis mouse models used in this report differed in regards to the effects of Aβ on IgM and IgG immunoglobulin levels. Possible explanations may emerge from a wider analysis of mice at different ages and may reflect fundamental differences in APP expression (i.e., endogenous mouse APP, humanized only with regard to the Aβ sequence, versus overexpression of the entire human APP coding sequence, as well transcription regulated by endogenous APP promoter versus transgene expression from the prion promoter) [12].

It will be critical to learn, which of the mechanisms of IgM depletion is at play in the *APP*^*NL-F/NL-F*^ mouse. Dependent on the underlying mechanism, this line of research may advance the goal to develop a blood-based AD diagnostic. Whereas it is likely that the relative IGHM depletion was caused by increased Aβ levels, other scenarios are also conceivable, including the possibility that the mutant APP parent molecule influences immune functions related to the secretion of IgMs. One way to test such an alternative scenario would be to compare IGHM levels in *APP*^*NL-F/NL-F*^ mice treated (or not) with inhibitors known to affect Aβ formation (e.g., directed against β-secretase).

Although countless prior attempts to detect blood-based AD biomarkers have failed, interest in identifying such a biomarker remains high because alternative strategies, including those relying on CSF sampling for ELISA analyses, or the delivery of positron emission tomography probes for brain imaging-based diagnostics are fraught with complex technical challenges of their own. Note that while bulk IgM depletion, which is also observed in SIgMD, may not be sufficiently selective to become useful as an AD diagnostic, the depletion of specific IgM subsets may have the necessary discriminatory power. A well-known disease whose diagnosis and etiology is linked to changes in auto-antibodies is lupus nephritis, which is characterized by high IgG levels directed against double-stranded DNA [43].

## Conclusions

The current study documented a surprising systemic depletion of IGHM levels in response to increased Aβ levels in a knock-in mouse model of AD-like amyloidosis. Urgently needed are follow-up experiments that (i) further validate this observation in other Aβ amyloidosis models, (ii) clarify the underlying mechanism(s) for selective IGHM targeting in these mice (e.g., can the IGHM depletion be blocked by inhibiting Aβ production?) and (iii) elucidate to what extent observations made in this study are transferable to human AD. The result of our study could accelerate translational research, because potentially it can be applied in early-intervention AD clinical trials, such as the Dominantly Inherited Alzheimer Network Trial (DIAN; http://clinicaltrials.gov/ct2/show/NCT01760005), for which IGHM levels might be evaluated in presymptomatic mutation carriers. Efforts invested in this direction may not only be of potential diagnostic value but may also shed light on the molecular biology that influences disease risk. In other words, although the depletion of IGHMs may be diagnostic for elevated Aβ1–42 in this model, it is conceivable that the organism’s resilience to counter this process may critically influence the risk of developing the disease.

## Supporting information

S1 Table Global proteome of *APP*^*NL-F*^ mouse brains at three ages. (XLSX)

S1 Fig Inconsistent effect of APP^*NL-F*^ genotype on IgM blood levels in littermates of mice younger than 15 months. (**a**) Comparison of IgM levels by Western blot analysis revealed that in younger (9- or 10- month-old) homozygous APP^*NL-F/NL-F*^ mice IgM levels in the blood exceeded those observed in age-matched wild-type (wt) or heterozygote APP^*NL-F/wt*^ mice. At intermediate ages (12- or 14-month-old) IgM levels were similar for all genotypes, and in 15-month-old mice relative IgM levels were lower in homozygous APP^*NL-F/NL-F*^ mice than in wild-type or heterozygous APP^*NL-F/wt*^ mice. (**b**) Western blot membrane shown in panel ‘a’ stained with Coomassie, documenting consistent overall protein levels in all blood samples analyzed. Littermates are grouped by black dotted boundaries.
(PDF)

S2 Fig Additional validation of reduced IgM levels in 15-month-old APP^*NL-F/NL-F*^ serum samples relative to APP^*NL-F/wt*^ heterozygous littermates. (**a**) Comparison of IgM levels by Western blot analysis revealed that in four out of six littermate pairings of heterozygous and homozygous APP^*NL-F*^ mice (indicated by black dotted boundaries), levels of IgM were relatively lower in the homozygous APP^*NL-F/NL-F*^ mice. The exception to this trend represented two pairings (indicated by blue dotted boundaries), collected from cages, in which mice were previously observed to exhibit increased hair loss. Note that the significance of this hair loss with regard to the molecular IgM phenotype analyzed in this experiment is currently obscure. (**b**) Western blot membrane shown in panel ‘a’ stained with Coomassie, documenting good agreement in levels of proteins for littermate pairings and lesser consistency of dominant serum protein signals for unpaired samples.
(PDF)

S3 Fig A tentative assessment of IGHM serum levels in CRND8 mice suggests that this model may not recapitulate the systemic depletion of this protein observed in the blood of *APP*^*NL-F/NL-F*^ homozygous mice. (**a**) Anti-IgM Western blot analysis of serum samples collected from 12-month-old wild-type (wt) mice or CRND8 transgenic littermates [22]. Although levels of IgM were not identical in the mice investigated, signal intensities of bands exhibited no apparent genotype correlation. (**b**) However, note the higher levels of IgG light chains in transgenic CRND8/wt mice relative to wt/wt littermates in Litter D housed in the same cage. Due to the small number of mice available for this pilot experiment, further work is needed to reveal the robustness of this observation. (**c**) Anti-Aβ Western blot analyses of brain homogenates validated genomic PCR-based genotyping results of mice with respect to the presence or absence of the human APP transgene array. Note the relative equal levels of transgene expression in the four mice, which had been predicted to carry the transgene array. For samples compared in the three Western blot panels depicted in this figure, an equal volume of serum was loaded in each lane and brain samples were adjusted for equal protein concentrations by bicinchoninic acid (BCA) assay.
(PDF)

S4 Fig Levels of phosphorylcholine-directed IgMs are not affected in serum of *APP*^*NL-F/NL-F*^ mice. (**a**) Calibration curve generated with anti-phosphorylcholine IgM ELISA kit. (**b**) Quantitation of (i) total IgM levels based on densitometric analysis of Western blot signals and (ii) anti-phosphorylcholine IgM levels based on ELISA measurements. The levels of phosphorylcholine-reactive IgM relative to total IgM was generally low at 15 months of age and did not seem to change when comparing wt/wt, NL-F/wt or NL-F/NL-F mice.
(PDF)

## Abbreviations

Aβ: amyloid β
AD: Alzheimer disease
ApoE: apolipoprotein E
APP: amyloid precursor protein
GO: gene ontology
IgG: immunoglobulin G
IGHM: immunoglobulin heavy mu chain
IgM: immunoglobulin M
IPI: international protein index
MCI: mild cognitive impairment
oxLDL: oxidized low density lipoprotein
PSM: peptide-to-spectrum matches
SDS: sodium dodecyl sulfate
SigMD: selective immunoglobulin M deficiency
TMT: tandem mass tag
wt: wild-type

## References

[1] Prince M, Wimo A, Guerchet M, Ali G-C, Wu Y-T, Prina M. World Alzheimer Report 2015: The Global Impact of Dementia, an analysis of prevalence, incidence, cost and trends. Alzheimer’s Disease International (ADI), London, August 2015. 2015.

[2] Glenner GG, Wong CW. Alzheimer's disease: initial report of the purification and characterization of a novel cerebrovascular amyloid protein. Biochem Biophys Res Commun. 1984;120(3):885–90. 6375662 10.1016/s0006-291x(84)80190-4

[3] Hardy JA, Higgins GA. Alzheimer's disease: the amyloid cascade hypothesis. Science. 1992;256(5054):184–5. 1566067 10.1126/science.1566067

[4] Corder EH, Saunders AM, Strittmatter WJ, Schmechel DE, Gaskell PC, Small GW, et al Gene dose of apolipoprotein E type 4 allele and the risk of Alzheimer's disease in late onset families. Science. 1993;261(5123):921–3. 8346443 10.1126/science.8346443

[5] Ghani M, Reitz C, St George-Hyslop P, Rogaeva E. Genetic Complexity of Early Onset Alzheimer’s disease In: Galimberti D, Scarpini E, editors. Neurodegenerative Diseases. London: Springer; 2017; in press.

[6] Chin J. Selecting a mouse model of Alzheimer's disease. Meth Mol Biol. 2011;670:169–89. 10.1007/978-1-60761-744-0_13 20967591

[7] Games D, Adams D, Alessandrini R, Barbour R, Berthelette P, Blackwell C, Carr T, et al Alzheimer-type neuropathology in transgenic mice overexpressing V717F beta-amyloid precursor protein. Nature. 1995;373(6514):523–7. doi: 10.1038/373523a0 7845465 10.1038/373523a0

[8] Hsiao K, Chapman P, Nilsen S, Eckman C, Harigaya Y, Younkin S, et al Correlative memory deficits, Abeta elevation, and amyloid plaques in transgenic mice. Science. 1996;274(5284):99–102. 8810256 10.1126/science.274.5284.99

[9] Sturchler-Pierrat C, Abramowski D, Duke M, Wiederhold KH, Mistl C, Rothacher S, et al Two amyloid precursor protein transgenic mouse models with Alzheimer disease-like pathology. Proc Natl Acad Sci USA. 1997;94(24):13287–92. 9371838 10.1073/pnas.94.24.13287 PMC24301

[10] Calhoun ME, Wiederhold KH, Abramowski D, Phinney AL, Probst A, Sturchler-Pierrat C, et al Neuron loss in APP transgenic mice. Nature. 1998;395(6704):755–6. doi: 10.1038/27351 9796810 10.1038/27351

[11] Bohm C, Chen F, Sevalle J, Qamar S, Dodd R, Li Y, Schmitt-Ulms G, et al Current and future implications of basic and translational research on amyloid-beta peptide production and removal pathways. Mol Cell Neurosci. 2015;66(Pt A):3–11. doi: 10.1016/j.mcn.2015.02.016 25748120 10.1016/j.mcn.2015.02.016 PMC4503820

[12] Saito T, Matsuba Y, Mihira N, Takano J, Nilsson P, Itohara S, et al Single App knock-in mouse models of Alzheimer's disease. Nat Neurosci. 2014;17(5):661–3. doi: 10.1038/nn.3697 24728269 10.1038/nn.3697

[13] Chishti MA, Yang DS, Janus C, Phinney AL, Horne P, Pearson J, et al Early-onset amyloid deposition and cognitive deficits in transgenic mice expressing a double mutant form of amyloid precursor protein 695. J Biol Chem. 2001;276(24):21562–70. doi: 10.1074/jbc.M100710200 11279122 10.1074/jbc.M100710200

[14] Mehrabian M, Brethour D, MacIsaac S, Kim JK, Gunawardana CG, Wang H, et al CRISPR-Cas9-Based Knockout of the Prion Protein and Its Effect on the Proteome. PLoS One. 2014;9(12):e114594 doi: 10.1371/journal.pone.0114594 25490046 10.1371/journal.pone.0114594 PMC4260877

[15] Gunawardana CG, Mehrabian M, Wang X, Mueller I, Lubambo IB, Jonkman JE, et al The human tau interactome: binding to the ribonucleoproteome, and impaired binding of the P301L mutant to chaperones and the proteasome. Mol Cell Proteomics. 2015;14(11):3000–14. doi: 10.1074/mcp.M115.050724 26269332 10.1074/mcp.M115.050724 PMC4638042

[16] Kall L, Canterbury JD, Weston J, Noble WS, MacCoss MJ. Semi-supervised learning for peptide identification from shotgun proteomics datasets. Nat Meth. 2007;4(11):923–5. 10.1038/nmeth1113 17952086

[17] Vizcaino JA, Csordas A, del-Toro N, Dianes JA, Griss J, Lavidas I, et al 2016 update of the PRIDE database and its related tools. Nucleic Acids Res. 2016;44(D1):D447–56. doi: 10.1093/nar/gkv1145 26527722 10.1093/nar/gkv1145 PMC4702828

[18] Schneider CA, Rasband WS, Eliceiri KW. NIH Image to ImageJ: 25 years of image analysis. Nat Meth. 2012;9(7):671–5. 10.1038/nmeth.2089 PMC5554542 22930834

[19] Cox J, Mann M. MaxQuant enables high peptide identification rates, individualized p.p.b.-range mass accuracies and proteome-wide protein quantification. Nat Biotech. 2008;26(12):1367–72. 10.1038/nbt.1511 19029910

[20] Dayon L, Sanchez JC. Relative protein quantification by MS/MS using the tandem mass tag technology. Meth Mol Biol. 2012;893:115–27. 10.1007/978-1-61779-885-6_9 22665298

[21] Lundgren DH, Hwang SI, Wu L, Han DK. Role of spectral counting in quantitative proteomics. Expert Rev Proteomics. 2010;7(1):39–53. doi: 10.1586/epr.09.69 20121475 10.1586/epr.09.69

[22] Janus C, Pearson J, McLaurin J, Mathews PM, Jiang Y, Schmidt SD, et al A beta peptide immunization reduces behavioural impairment and plaques in a model of Alzheimer's disease. Nature. 2000;408(6815):979–82. 10.1038/35050110 11140685

[23] Song F, Poljak A, Kochan NA, Raftery M, Brodaty H, Smythe GA, et al Plasma protein profiling of Mild Cognitive Impairment and Alzheimer's disease using iTRAQ quantitative proteomics. Proteome Sci. 2014;12(1):5 doi: 10.1186/1477-5956-12-5 24433274 10.1186/1477-5956-12-5 PMC3898732

[24] Inoue T, Okumura Y, Shirama M, Ishibashi H, Kashiwagi S, Okubo H. Selective partial IgM deficiency: functional assessment of T and B lymphocytes in vitro. J Clin Immunol. 1986;6(2):130–5. 2872228 10.1007/BF00918745

[25] Shaw PX, Horkko S, Chang MK, Curtiss LK, Palinski W, Silverman GJ, et al Natural antibodies with the T15 idiotype may act in atherosclerosis, apoptotic clearance, and protective immunity. J Clin Invest. 2000;105(12):1731–40. doi: 10.1172/JCI8472 10862788 10.1172/JCI8472 PMC378505

[26] deCathelineau AM, Henson PM. The final step in programmed cell death: phagocytes carry apoptotic cells to the grave. Essays Biochem. 2003;39:105–17. 14585077 10.1042/bse0390105

[27] Chou MY, Fogelstrand L, Hartvigsen K, Hansen LF, Woelkers D, Shaw PX, et al Oxidation-specific epitopes are dominant targets of innate natural antibodies in mice and humans. J Clin Invest. 2009;119(5):1335–49. doi: 10.1172/JCI36800 19363291 10.1172/JCI36800 PMC2673862

[28] Eriksson UK, Sjoberg BG, Bennet AM, de Faire U, Pedersen NL, Frostegard J. Low levels of antibodies against phosphorylcholine in Alzheimer's disease. J Alzheim Dis. 2010;21(2):577–84. 10.3233/JAD-2010-091705 20571218

[29] Silajdzic E, Bjorkqvist M, Hansson O. Antibodies against phosphorylcholine are not altered in plasma of patients with Alzheimer's disease. BMC Neurol. 2015;15:8 doi: 10.1186/s12883-015-0260-1 25651913 10.1186/s12883-015-0260-1 PMC4324431

[30] Gaskin F, Finley J, Fang Q, Xu S, Fu SM. Human antibodies reactive with beta-amyloid protein in Alzheimer's disease. J Exp Med. 1993;177(4):1181–6. 8459212 10.1084/jem.177.4.1181 PMC2190957

[31] Kawarabayashi T, Shoji M. Plasma biomarkers of Alzheimer's disease. Curr Opin Psychiatry. 2008;21(3):260–7. doi: 10.1097/YCO.0b013e3282fc989f 18382225 10.1097/YCO.0b013e3282fc989f

[32] Li Q, Gordon M, Cao C, Ugen KE, Morgan D. Improvement of a low pH antigen-antibody dissociation procedure for ELISA measurement of circulating anti-Abeta antibodies. BMC Neurosci. 2007;8:22 doi: 10.1186/1471-2202-8-22 17374155 10.1186/1471-2202-8-22 PMC1845169

[33] Gustaw-Rothenberg KA, Siedlak SL, Bonda DJ, Lerner A, Tabaton M, Perry G, et al Dissociated amyloid-beta antibody levels as a serum biomarker for the progression of Alzheimer's disease: a population-based study. Exp Gerontol. 2010;45(1):47–52. doi: 10.1016/j.exger.2009.10.003 19819324 10.1016/j.exger.2009.10.003 PMC2815174

[34] Marcello A, Wirths O, Schneider-Axmann T, Degerman-Gunnarsson M, Lannfelt L, Bayer TA. Circulating immune complexes of Abeta and IgM in plasma of patients with Alzheimer's disease. J Neural Transm. 2009;116(7):913–20. doi: 10.1007/s00702-009-0224-y 19415450 10.1007/s00702-009-0224-y PMC2700872

[35] Marcello A, Wirths O, Schneider-Axmann T, Degerman-Gunnarsson M, Lannfelt L, Bayer TA. Reduced levels of IgM autoantibodies against N-truncated pyroglutamate Abeta in plasma of patients with Alzheimer's disease. Neurobiol Aging. 2011;32(8):1379–87. doi: 10.1016/j.neurobiolaging.2009.08.011 19781815 10.1016/j.neurobiolaging.2009.08.011

[36] Giedraitis V, Sundelof J, Irizarry MC, Garevik N, Hyman BT, Wahlund LO, et al The normal equilibrium between CSF and plasma amyloid beta levels is disrupted in Alzheimer's disease. Neurosci Lett. 2007;427(3):127–31. doi: 10.1016/j.neulet.2007.09.023 17936506 10.1016/j.neulet.2007.09.023

[37] Moir RD, Tseitlin KA, Soscia S, Hyman BT, Irizarry MC, Tanzi RE. Autoantibodies to redox-modified oligomeric Abeta are attenuated in the plasma of Alzheimer's disease patients. J Biol Chem. 2005;280(17):17458–63. doi: 10.1074/jbc.M414176200 15728175 10.1074/jbc.M414176200

[38] Lindhagen-Persson M, Brannstrom K, Vestling M, Steinitz M, Olofsson A. Amyloid-beta oligomer specificity mediated by the IgM isotype—implications for a specific protective mechanism exerted by endogenous auto-antibodies. PLoS One. 2010;5(11):e13928 doi: 10.1371/journal.pone.0013928 21085663 10.1371/journal.pone.0013928 PMC2978096

[39] Pihlgren M, Silva AB, Madani R, Giriens V, Waeckerle-Men Y, Fettelschoss A, et al TLR4- and TRIF-dependent stimulation of B lymphocytes by peptide liposomes enables T cell-independent isotype switch in mice. Blood. 2013;121(1):85–94. doi: 10.1182/blood-2012-02-413831 23144170 10.1182/blood-2012-02-413831

[40] Tahara K, Kim HD, Jin JJ, Maxwell JA, Li L, Fukuchi K. Role of toll-like receptor signalling in Abeta uptake and clearance. Brain. 2006;129(Pt 11):3006–19. doi: 10.1093/brain/awl249 16984903 10.1093/brain/awl249 PMC2445613

[41] Zhang W, Wang LZ, Yu JT, Chi ZF, Tan L. Increased expressions of TLR2 and TLR4 on peripheral blood mononuclear cells from patients with Alzheimer's disease. J Neurol Sci. 2012;315(1–2):67–71. doi: 10.1016/j.jns.2011.11.032 22166855 10.1016/j.jns.2011.11.032

[42] Udan ML, Ajit D, Crouse NR, Nichols MR. Toll-like receptors 2 and 4 mediate Abeta(1–42) activation of the innate immune response in a human monocytic cell line. J Neurochem. 2008;104(2):524–33. doi: 10.1111/j.1471-4159.2007.05001.x 17986235 10.1111/j.1471-4159.2007.05001.x

[43] Yung S, Chan TM. Autoantibodies and resident renal cells in the pathogenesis of lupus nephritis: getting to know the unknown. Clin Dev Immunol. 2012;2012:139365 doi: 10.1155/2012/139365 22761629 10.1155/2012/139365 PMC3386553

