# Supplementary figures and images for: Time-course global proteome analyses reveal an inverse correlation between Aβ burden and immunoglobulin M levels in the APPNL-F mouse model of Alzheimer disease
Source: PLoS One. 2017 Aug 23;12(8):e0182844. doi: 10.1371/journal.pone.0182844 (PMC5568403; doi:10.1371/journal.pone.0182844)

**S1 Fig.**

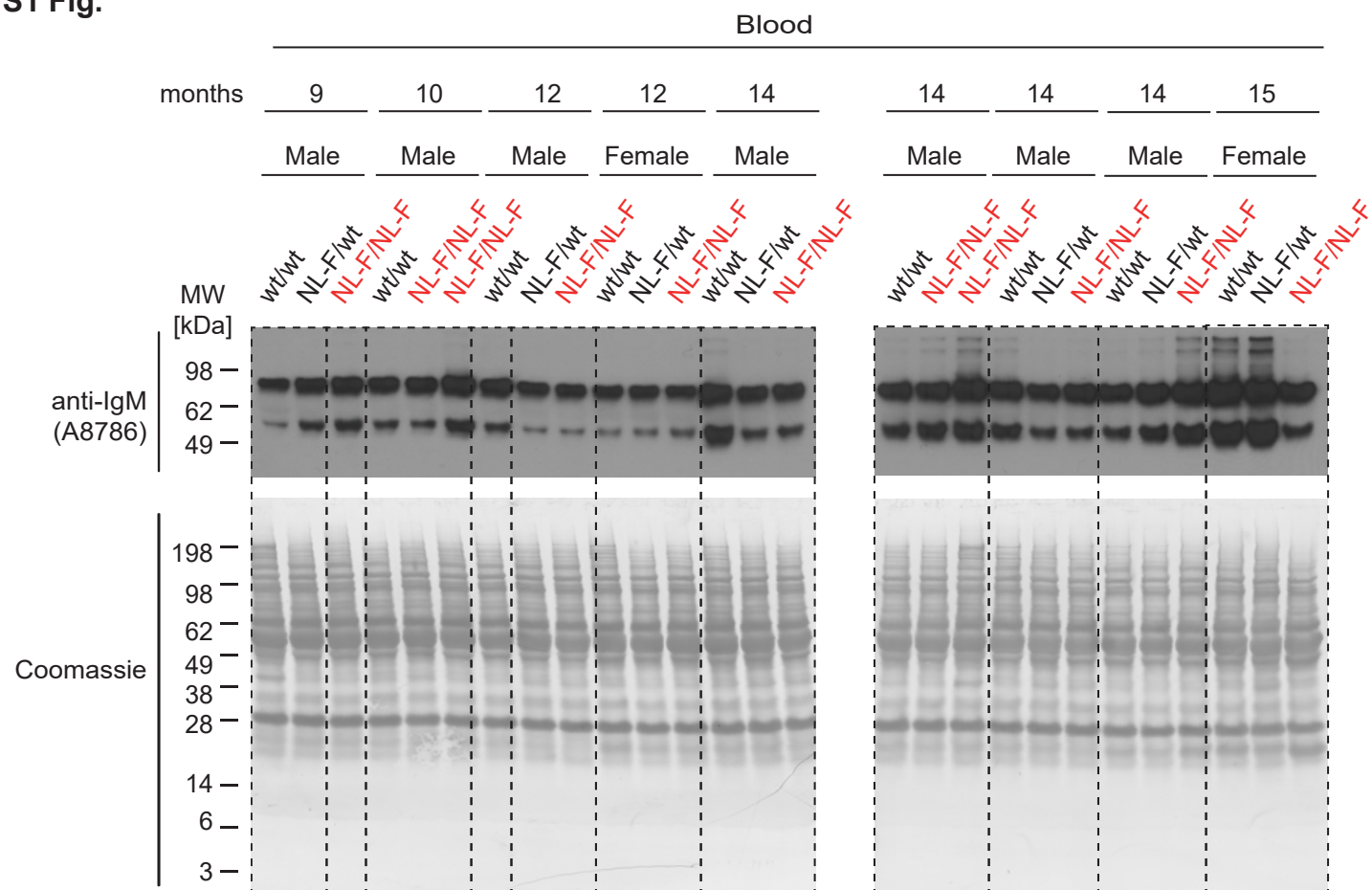

Supplement: S1 Fig — (a) Comparison of IgM levels by Western blot analysis revealed that in younger (9- or 10- month-old) homozygous APPNL-F/NL-F mice IgM levels in the blood exceeded those observed in age-matched wild-type (wt) or heterozygote APPNL-F/wt mice. At intermediate ages (12- or 14-month-old) IgM levels were similar for all genotypes, and in 15-month-old mice relative IgM levels were lower in homozygous APPNL-F/NL-F mice than in wild-type or heterozygous APPNL-F/wt mice. (b) Western blot membrane shown in panel ‘a’ stained with Coomassie, documenting consistent overall protein levels in all blood samples analyzed. Littermates are grouped by black dotted boundaries. (PDF) [file pone.0182844.s002.pdf]

S2 Fig.

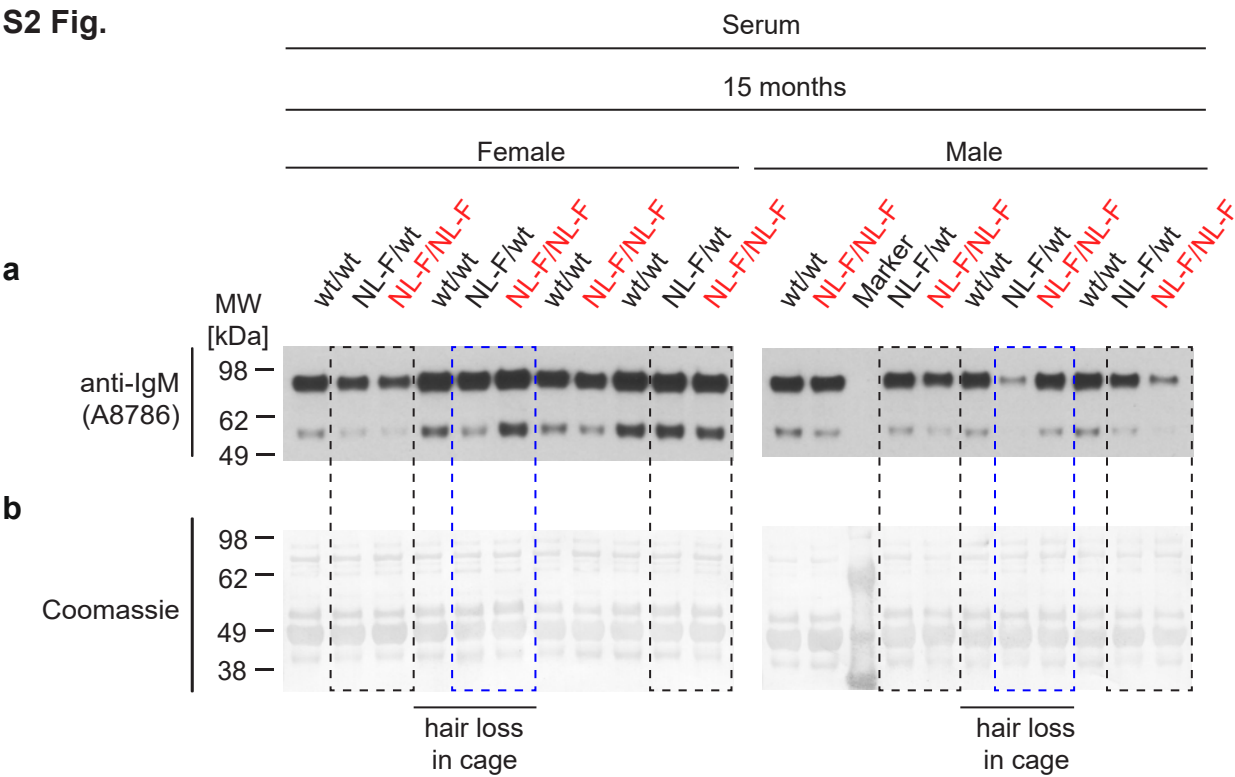

Supplement: S2 Fig — (a) Comparison of IgM levels by Western blot analysis revealed that in four out of six littermate pairings of heterozygous and homozygous APPNL-F mice (indicated by black dotted boundaries), levels of IgM were relatively lower in the homozygous APPNL-F/NL-F mice. The exception to this trend represented two pairings (indicated by blue dotted boundaries), collected from cages, in which mice were previously observed to exhibit increased hair loss. Note that the significance of this hair loss with regard to the molecular IgM phenotype analyzed in this experiment is currently obscure. (b) Western blot membrane shown in panel ‘a’ stained with Coomassie, documenting good agreement in levels of proteins for littermate pairings and lesser consistency of dominant serum protein signals for unpaired samples. (PDF) [file pone.0182844.s003.pdf]

S3 Fig.

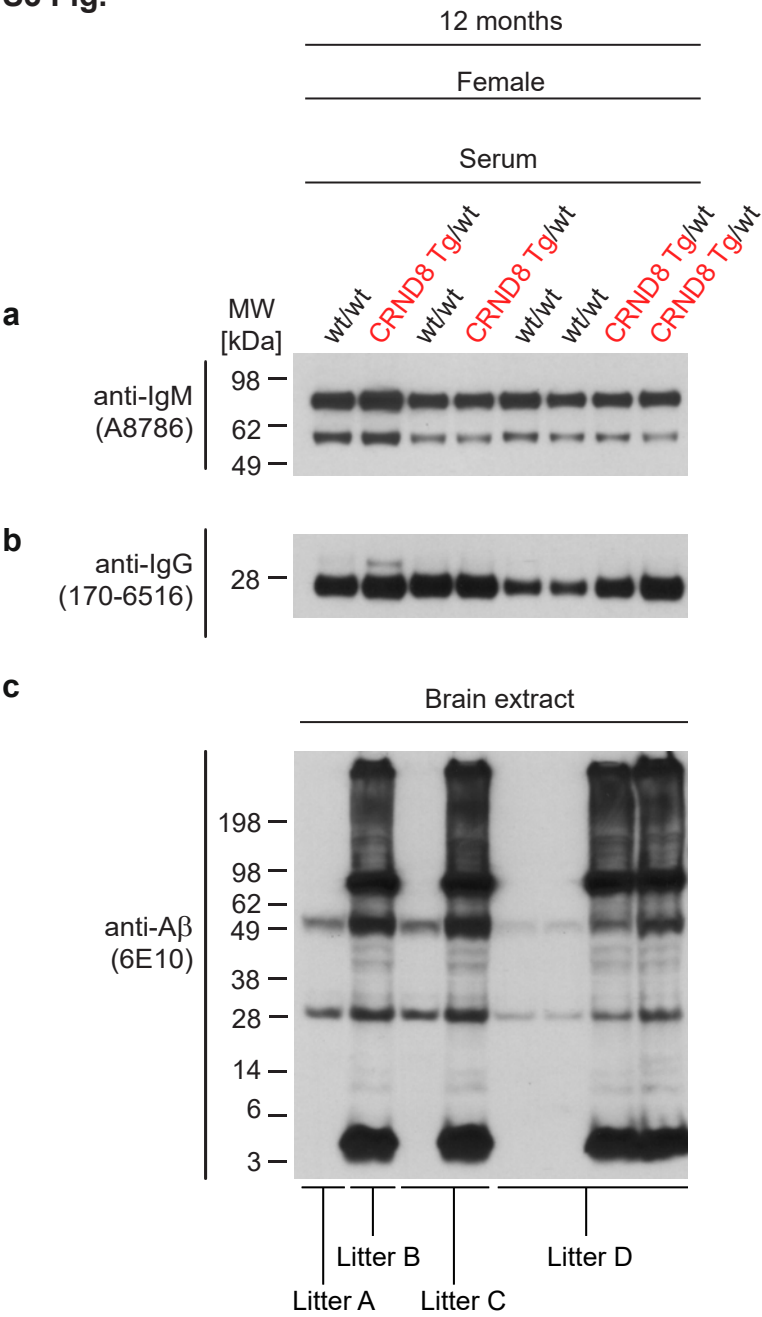

Supplement: S3 Fig — (a) Anti-IgM Western blot analysis of serum samples collected from 12-month-old wild-type (wt) mice or CRND8 transgenic littermates [22]. Although levels of IgM were not identical in the mice investigated, signal intensities of bands exhibited no apparent genotype correlation. (b) However, note the higher levels of IgG light chains in transgenic CRND8/wt mice relative to wt/wt littermates in Litter D housed in the same cage. Due to the small number of mice available for this pilot experiment, further work is needed to reveal the robustness of this observation. (c) Anti-Aβ Western blot analyses of brain homogenates validated genomic PCR-based genotyping results of mice with respect to the presence or absence of the human APP transgene array. Note the relative equal levels of transgene expression in the four mice, which had been predicted to carry the transgene array. For samples compared in the three Western blot panels depicted in this figure, an equal volume of serum was loaded in each lane and brain samples were adjusted for equal protein concentrations by bicinchoninic acid (BCA) assay. (PDF) [file pone.0182844.s004.pdf]

S4 Fig.

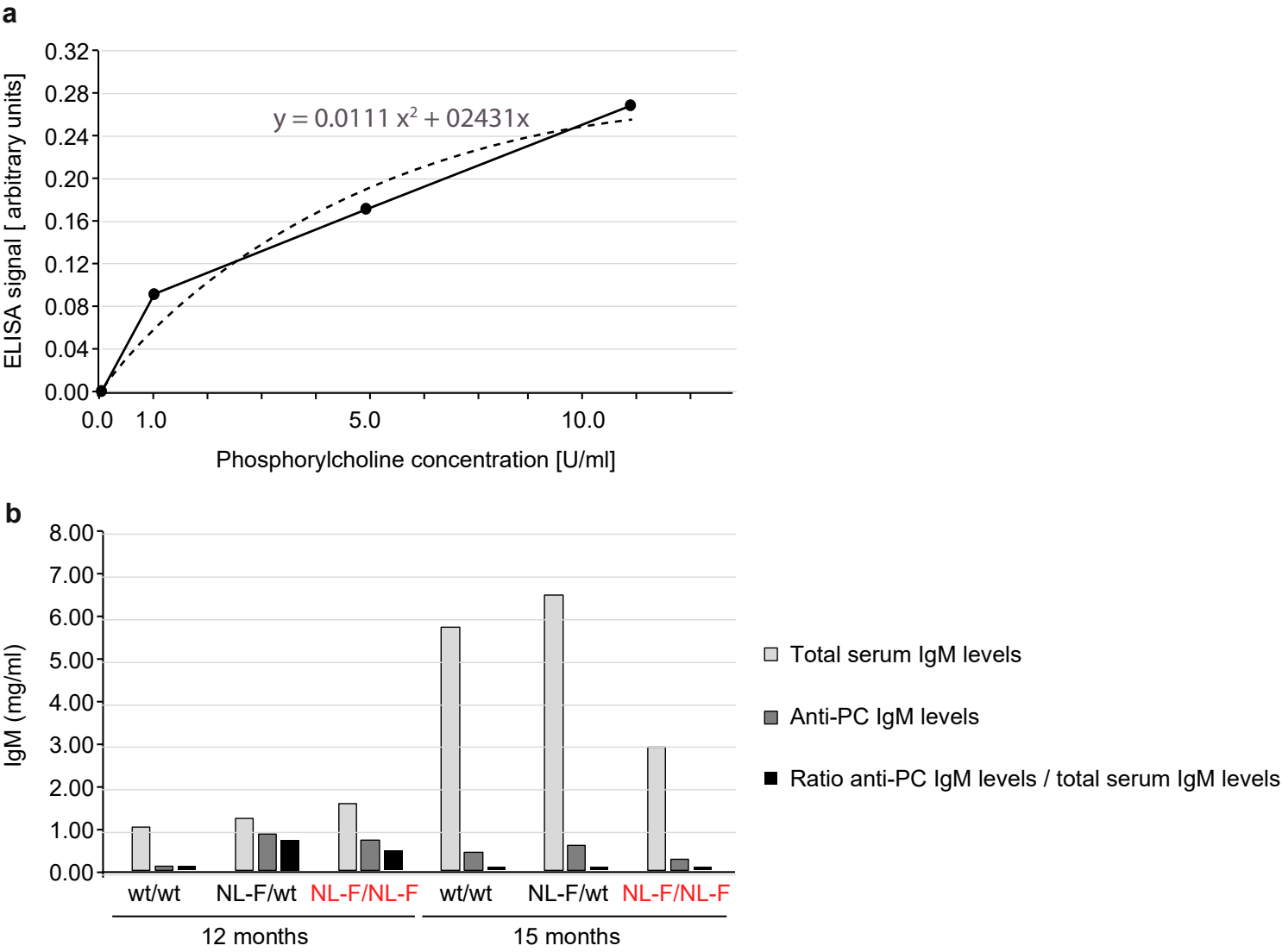

Supplement: S4 Fig — (a) Calibration curve generated with anti-phosphorylcholine IgM ELISA kit. (b) Quantitation of (i) total IgM levels based on densitometric analysis of Western blot signals and (ii) anti-phosphorylcholine IgM levels based on ELISA measurements. The levels of phosphorylcholine-reactive IgM relative to total IgM was generally low at 15 months of age and did not seem to change when comparing wt/wt, NL-F/wt or NL-F/NL-F mice. (PDF) [file pone.0182844.s005.pdf]
